# Blood pressure-lowering treatment for the prevention of cardiovascular events in patients with atrial fibrillation: An individual participant data meta-analysis

**DOI:** 10.1371/journal.pmed.1003599

**Published:** 2021-06-01

**Authors:** Ana-Catarina Pinho-Gomes, Luis Azevedo, Emma Copland, Dexter Canoy, Milad Nazarzadeh, Rema Ramakrishnan, Eivind Berge, Johan Sundström, Dipak Kotecha, Mark Woodward, Koon Teo, Barry R. Davis, John Chalmers, Carl J. Pepine, Kazem Rahimi, on behalf of the Blood Pressure Lowering Treatment Trialists’ Collaboration

**Affiliations:** 1 Faculty of Life Sciences and Medicine, King’s College London, London, United Kingdom; 2 Department of Community Medicine, Information and Health Decision Sciences, Centre for Health Technology and Services Research, Faculty of Medicine, University of Porto, Porto, Portugal; 3 Deep Medicine, Nuffield Department of Women’s and Reproductive Health, University of Oxford, Oxford, United Kingdom; 4 National Institute for Health Research Oxford Biomedical Research Centre, Oxford University Hospitals National Health Service Foundation Trust, Oxford, United Kingdom; 5 Department of Cardiology, Oslo University Hospital, Oslo, Norway; 6 Institute for Clinical Medicine, University of Tromsø, Norway; 7 Department of Medical Sciences, Uppsala University, Sweden; 8 Institute of Cardiovascular Sciences, University of Birmingham, Birmingham, United Kingdom; 9 The George Institute for Global Health, University of New South Wales, Sydney, Australia; 10 The George Institute for Global Health, Department of Epidemiology and Biostatistics, Imperial College, London, United Kingdom; 11 Department of Epidemiology, Johns Hopkins University, Baltimore, Maryland, United States of America; 12 Population Health Research Institute, McMaster University, Hamilton, Ontario, Canada; 13 The University of Texas School of Public Health, Houston, Texas, United States of America; 14 Department of Medicine, University of Florida, Gainesville, Florida, United States of America; University of Liverpool, UNITED KINGDOM

## Abstract

**Background:**

Randomised evidence on the efficacy of blood pressure (BP)-lowering treatment to reduce cardiovascular risk in patients with atrial fibrillation (AF) is limited. Therefore, this study aimed to compare the effects of BP-lowering drugs in patients with and without AF at baseline.

**Methods and findings:**

The study was based on the resource provided by the Blood Pressure Lowering Treatment Trialists’ Collaboration (BPLTTC), in which individual participant data (IPD) were extracted from trials with over 1,000 patient-years of follow-up in each arm, and that had randomly assigned patients to different classes of BP-lowering drugs, BP-lowering drugs versus placebo, or more versus less intensive BP-lowering regimens. For this study, only trials that had collected information on AF status at baseline were included. The effects of BP-lowering treatment on a composite endpoint of major cardiovascular events (stroke, ischaemic heart disease or heart failure) according to AF status at baseline were estimated using fixed-effect one-stage IPD meta-analyses based on Cox proportional hazards models stratified by trial. Furthermore, to assess whether the associations between the intensity of BP reduction and cardiovascular outcomes are similar in those with and without AF at baseline, we used a meta-regression. From the full BPLTTC database, 28 trials (145,653 participants) were excluded because AF status at baseline was uncertain or unavailable. A total of 22 trials were included with 188,570 patients, of whom 13,266 (7%) had AF at baseline. Risk of bias assessment showed that 20 trials were at low risk of bias and 2 trials at moderate risk. Meta-regression showed that relative risk reductions were proportional to trial-level intensity of BP lowering in patients with and without AF at baseline. Over 4.5 years of median follow-up, a 5-mm Hg systolic BP (SBP) reduction lowered the risk of major cardiovascular events both in patients with AF (hazard ratio [HR] 0.91, 95% confidence interval [CI] 0.83 to 1.00) and in patients without AF at baseline (HR 0.91, 95% CI 0.88 to 0.93), with no difference between subgroups. There was no evidence for heterogeneity of treatment effects by baseline SBP or drug class in patients with AF at baseline. The findings of this study need to be interpreted in light of its potential limitations, such as the limited number of trials, limitation in ascertaining AF cases due to the nature of the arrhythmia and measuring BP in patients with AF.

**Conclusions:**

In this meta-analysis, we found that BP-lowering treatment reduces the risk of major cardiovascular events similarly in individuals with and without AF. Pharmacological BP lowering for prevention of cardiovascular events should be recommended in patients with AF.

## Abstract 2

In an individual patient data meta-analysis, Ana-Catarina Pinho-Gomes and colleagues investigate prevention of cardiovascular events with blood pressure-lowering treatment in those with and without atrial fibrillation.

## Author summary

**Why was this study done?:**

- Atrial fibrillation (AF) is the most common cardiac arrhythmia across the world and is strongly associated with future vascular disease, particularly stroke.
- Blood pressure (BP) lowering is an established strategy for prevention of vascular disease, but whether patients with AF benefit similarly from pharmacological BP reduction is not well understood.

**What did the researchers do and find?:**

- We compared the preventive effect of BP-lowering treatment on cardiovascular outcomes in patients with and without AF at baseline.
- We conducted an individual participant data meta-analysis using published and unpublished data from large randomised clinical trials (22 trials involving 188,570 patients).
- We showed that BP-lowering treatment reduced the risk of a major cardiovascular events with no evidence that effects differed according to the presence or absence of AF at baseline.
- The relative risk reductions were proportional to the intensity of BP reduction in individuals with and without AF.
- In individuals with AF, the relative risk reduction was comparable irrespective of whether baseline systolic BP was under or over the conventional treatment threshold of 140 mm Hg.

**What do these findings mean?:**

- BP-lowering treatment reduces the risk of major cardiovascular events in patients with AF to a similar extent to that of patients without AF.
- Pharmacological BP-lowering treatment for prevention of cardiovascular events should be recommended as part of care for patients with AF.

## Introduction

Atrial fibrillation (AF) is the most common clinically relevant cardiac arrhythmia and its incidence and prevalence are on the rise across the globe [1,2], mainly due to population ageing and an increase in other cardiometabolic risk factors [3]. In observational studies, AF has been associated with an approximately 90% higher risk of a fatal vascular event, such as stroke, ischaemic heart disease, heart failure (HF), and vascular dementia [4]. Although the risk of stroke, in particular, can be mitigated by anticoagulation, the majority of deaths in contemporary anticoagulated AF patients are due to cardiovascular causes other than stroke, such as myocardial infarction and HF [5,6]. Yet, there is no proven pharmacological intervention other than anticoagulation for effective reduction of such risks [7].

Although high blood pressure (BP) is the most common cardiovascular risk factor in patients with AF [8,9], whether BP lowering reduces the risk of cardiovascular events in patients with AF remains uncertain. As BP-lowering treatment significantly decreases cardiovascular risk in high-risk populations [10], a similar effect could be expected in patients with preexisting AF. However, the complex structural, neurohumoral, and metabolic changes in the cardiovascular system that underpin the development and progression of AF may interfere with BP-lowering treatment [11]. This uncertainty is further compounded by the fact that the only randomised controlled trial (RCT) specifically conducted in patients with AF failed to detect a risk reduction in cardiovascular events using an angiotensin receptor blocker [12]. Several other major BP-lowering trials have included patients with known AF, but the low prevalence of AF rendered them individually underpowered to perform subgroup analysis according to AF status at baseline. We have sought to extract previously published and unpublished data to compare the effect of BP-lowering treatment on fatal and nonfatal cardiovascular outcomes in patients with and without AF overall and by major drug classes.

## Methods

### Study design

We conducted individual participant data (IPD) meta-analyses of BP-lowering RCTs that investigated treatment effects on cardiovascular outcomes by presence or absence of AF at randomisation. The study was based on the resource provided by the Blood Pressure Lowering Treatment Trialists’ Collaboration (BPLTTC). RCTs are eligible for inclusion in BPLTTC if they have randomised participants to BP-lowering drugs versus placebo or alternative classes of BP-lowering drugs, or between more versus less intensive regimens, and have at least 1,000 patient-years of follow-up in each randomised arm. To date, 50 RCTs have shared data. Details of the methods underlying the latest cycle of the BPLTTC have been recently published and are described in S1 Methods [13]. A separate ethical approval was not required for this study. This analysis followed a prespecified protocol that is available as Supporting information (S1 Protocol).

In this study, only trials that had collected information on AF status at baseline were included. Three types of trials were identified: (1) trials that included both patients with and without AF at baseline; (2) trials that included only patients with AF at baseline; and (3) trials that excluded patients with AF at baseline. We excluded trials in which the presence of AF was not explicitly assessed at baseline or in which AF status at baseline was not clear.

### Definition of outcomes

The primary outcome was total cardiovascular events, defined as the first occurrence of (1) fatal or nonfatal stroke; (2) fatal or nonfatal myocardial infarction or ischaemic heart disease; or (3) HF causing death or requiring hospitalisation. Secondary outcomes were the individual elements of the composite endpoint as well as cardiovascular death and all-cause death.

### Treatment comparisons

For the main analysis, intervention and control groups were compared. For placebo-controlled trials, the placebo arm was considered as the “comparator” and the active treatment was considered as the “intervention.” For trials with two or more active treatment arms, the arm in which the BP reduction was higher was considered as “intervention” and the other treatment arm(s) as “comparator.” Treatment arms were grouped together whenever required to avoid double counting of participants. S1 Table summarises the treatment comparisons considered in each trial and the difference in systolic BP (SBP) reduction between trial arms.

### Risk of bias assessment

We used the Rob2 tool from the Cochrane Collaboration for assessing risk of bias of individual trials. (S2 Table) [14].

### Statistical analysis

Our main analyses aimed to address 4 questions: (q1) whether the effect of BP-lowering treatment on CVD outcomes differs between those with and without AF; (q2) whether the associations between the intensity of BP reduction and outcomes are similar in those with and without AF at baseline; (q3) whether in patients with AF, treatment effects vary by baseline SBP; and (q4) whether in patients with AF, treatment effects vary by classes of antihypertensives. Intention-to-treat analysis was adopted using the data provided by each trial, after internal quality checks had been carried out to ensure that data were accurate and transferred without error. Our method for investigating these questions was a one-stage approach that uses IPD from all trials simultaneously and applying a single statistical model. The one-stage approach has more power and flexibility than a two-stage approach to test for treatment-covariate interactions even when few studies are available [15,16]. We used fixed-effect one-stage IPD meta-analysis models for time-to-event data by applying Cox proportional hazard models stratified by trial [17]. The average SBP reduction between arms among all included trials was 3.7 mm Hg (due to inclusion of “head-to-head” comparisons trials) (S1 Table). Thus, we adjusted the estimates for each subgroup (with and without AF) for a 5-mm Hg reduction in SBP. Furthermore, to assess whether the associations between the intensity of BP reduction and cardiovascular outcomes is similar in those with and without AF at baseline (q2), we used analytical and graphical representations of the full meta-regression model with additional terms for AF status and interactions between treatment, difference in SBP, and AF status. This model describes the effects on outcomes for each level of intensity of SBP lowering and for each of the subgroups with and without AF at baseline. Finally, to assess in patients with AF whether treatment effects vary by baseline SBP and by classes of BP-lowering drugs (q3 and q4), we used models only for AF patients with additional terms for these potential moderators and interactions between treatment, difference in SBP, and moderators (S3 and S4 Tables). Further details on our statistical modelling approach, subgroup analyses, and sensitivity analyses are provided in S1 Methods. Statistical analyses were performed using R version 3.6.1. This study is reported as per the Preferred Reporting Items for Systematic Reviews and Meta-Analyses (PRISMA) guideline (S1 PRISMA Checklist).

## Results

**Fig 1:**
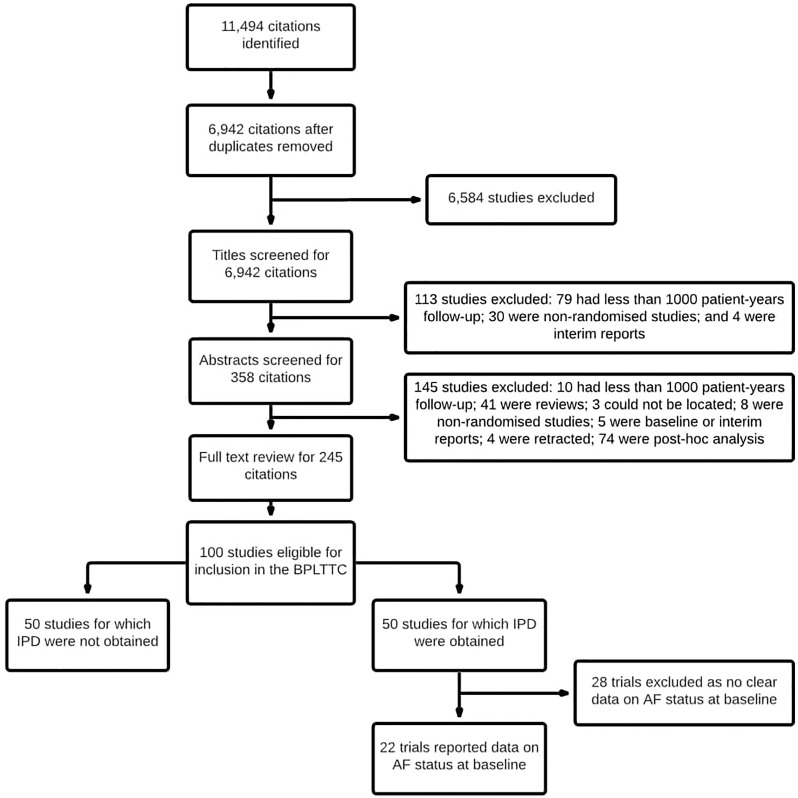
PRISMA diagram for included trials. AF, atrial fibrillation; BPLTTC, Blood Pressure Lowering Treatment Trialists’ Collaboration; IPD, individual participant data; PRISMA, Preferred Reporting Items for Systematic Reviews and Meta-Analyses.

From the full BPLTTC database, 28 trials (145,653 participants) were excluded because AF status at baseline was uncertain or unavailable. Twenty-two trials were eligible and provided data for the IPD meta-analyses (S5 and S6 Tables and Fig 1). Seven of these trials had previously published data about AF status at baseline. The 22 trials included 188,570 individuals, of whom 13,266 (7%) had AF at baseline [12,18–33]. Seven trials explicitly excluded participants with AF at baseline (*N =* 13,170, 7% of the participants without AF at baseline) [21,34–37], and one trial included only those with prevalent AF (*N* = 9,016, 67% of the participants with AF at baseline) [12]. The remaining 14 trials included a mixed population of participants with and without AF at baseline (4,249 with AF and 153,198 without AF). All trials contributed data for all the outcomes of interest, with the exception of 2 trials that did not report the HF outcome [19,23] and one trial that did not report cardiovascular death [35]. Risk of bias assessment showed that 20 trials had low risk of bias and 2 trials had moderate risk (S2 Table). There was no evidence of acquisition bias based on funnel plot and Egger’s regression test (S1 Fig).

Patients with AF were older than those without AF (mean age 70 versus 65 years, respectively) (Table 1). A lower baseline SBP and diastolic BP (DBP) was evident in patients with AF, who were more commonly prescribed diuretics, angiotensin-converting enzyme inhibitors, beta-blockers, and alpha-blockers: 143/84 mm Hg (SD 21/12 mm Hg) versus 155/88 mm Hg (SD 21/13 mm Hg) in patients without AF, respectively. Cerebrovascular disease was more common in patients with AF, while ischaemic heart disease, diabetes mellitus, and chronic kidney disease were more common in patients without AF. The prevalence of smoking was higher in patients with AF than in those without AF (9.3% versus 24.3%, respectively).

**Table 1:** Baseline characteristics of participants by atrial fibrillation status at baseline.

	Atrial fibrillation (*N =* 13,266)	No atrial fibrillation (*N* = 175,304)	Total (*N* = 188,570)
Age (years)	70.19 (9.12)	65.36 (9.05)	65.70 (9.14)
Sex (Female)	5,052 (38.1)	73,182 (41.7)	78,235 (41.5)
Ischaemic heart disease	3,923 (29.6)	56,759 (32.4)	60,682 (32.2)
Cerebrovascular disease	2,395 (18.5)	21,788 (15.5)	24,183 (15.7)
Diabetes mellitus	3,569 (26.9)	54,209 (32.2)	57,778 (31.8)
Chronic kidney disease	163 (20.3)	15,600 (25.4)	15,763 (25.3)
Heart failure	2,882 (31.9)	0 (0)	2,882 (31.9)
Smoking (current)	1,224 (9.3)	38,283 (24.3)	39,507 (23.1)
Body mass index (kg/m^2^)	28.78 (5.60)	28.12 (9.67)	28.16 (9.44)
Total cholesterol (mmol/L)	5.3 (1.2)	5.6 (1.2)	5.6 (1.2)
Systolic blood pressure (mm Hg)	142.8 (20.9)	154.6 (21.6)	153.82 (21.72)
Diastolic blood pressure (mm Hg)	83.6 (11.7)	87.8 (12.6)	87.49 (12.58)
Pharmacological treatment			
Diuretic	6,082 (50.8)	19,196 (23.8)	25,278 (27.3)
Alpha-blocker	1,134 (10.7)	2,813 (4.4)	3,947 (5.2)
Beta-blocker	6,133 (51.3)	29,013 (36.0)	35,146 (38.0)
Angiotensin-converting enzyme inhibitor	6,846 (59.6)	32,290 (44.0)	39,136 (46.1)
Angiotensin receptor blocker	568 (5.4)	6,420 (15.0)	6,988 (13.1)
Calcium channel blocker	3,557 (29.7)	29,566 (36.7)	33,123 (35.8)
Anticoagulant	4,418 (37.8)	1,823 (3.1)	6,241 (8.9)
Antiplatelet	6,443 (56.1)	35,539 (49.3)	41,982 (50.2)
Lipid-lowering drug	3,742 (32.8)	31,100 (42.6)	34,842 (41.3)

All categorical variables are summarised as N (% yes); all continuous variables as mean (standard deviation).

In placebo-controlled trials (8 trials), the difference in SBP reduction between arms was 7.2 (SD 3.9) mm Hg; in drug–drug comparisons (12 trials), it was 2.3 (SD 0.9) mm Hg; and in more versus less intensive BP lowering (2 trials), it was 10.9 (SD 3.0) mm Hg. Overall, the mean difference in SBP reduction between intervention and control arms was 3.7 (SD 3.2) mm Hg, and that was similar in patients with and without AF (3.3 (SD 2.0) mm Hg versus 3.7 (SD 3.3) mm Hg for patients with and without AF, respectively).

**Fig 2:**
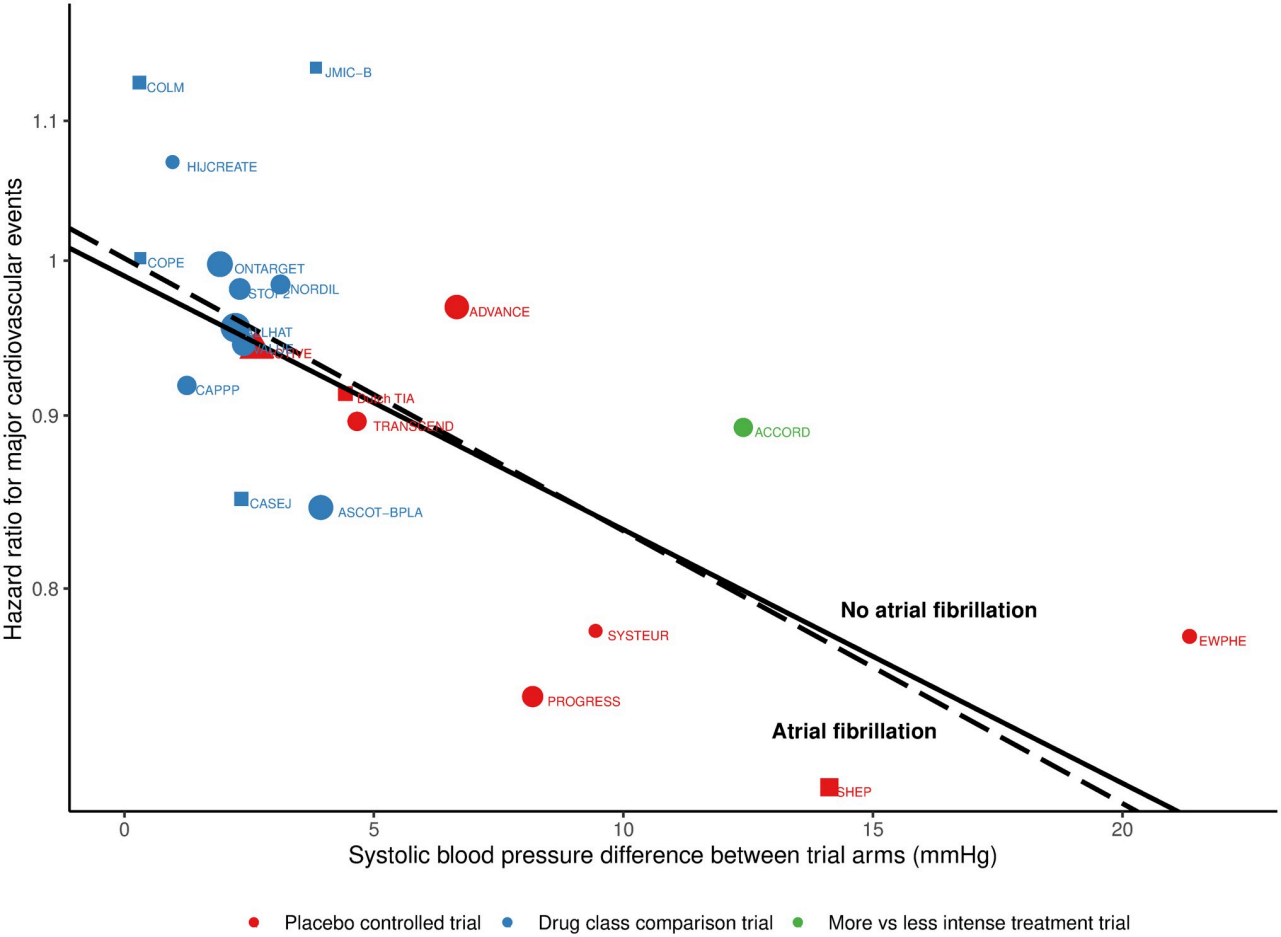
Hazard ratio of major cardiovascular events related to the 1-year difference blood pressure reduction aggregated at trial level. Risk of major cardiovascular events, for patients with (dashed line) and without (solid line) atrial fibrillation at baseline, regressed against the systolic blood pressure difference between trial arms, plotted on the log scale. Shapes represent the hazard ratio for each trial with the size inversely proportional to the respective standard error. Trials are coded by shape according to the type of patients: atrial fibrillation only (triangle), no atrial fibrillation only (square), and mix of both (circles). Trials are also coded by colour according to type of intervention: placebo-controlled trials (red), drug class comparison trials (blue), and more versus less intense treatment trials (green). Systolic blood pressure difference between trial arms in mm Hg.

Meta-regression showed that there was a linear association between the degree of SBP lowering and the reduction in the hazard ratio (HR) for major cardiovascular events both in patients with and without AF at baseline (Fig 2).

**Fig 3:**
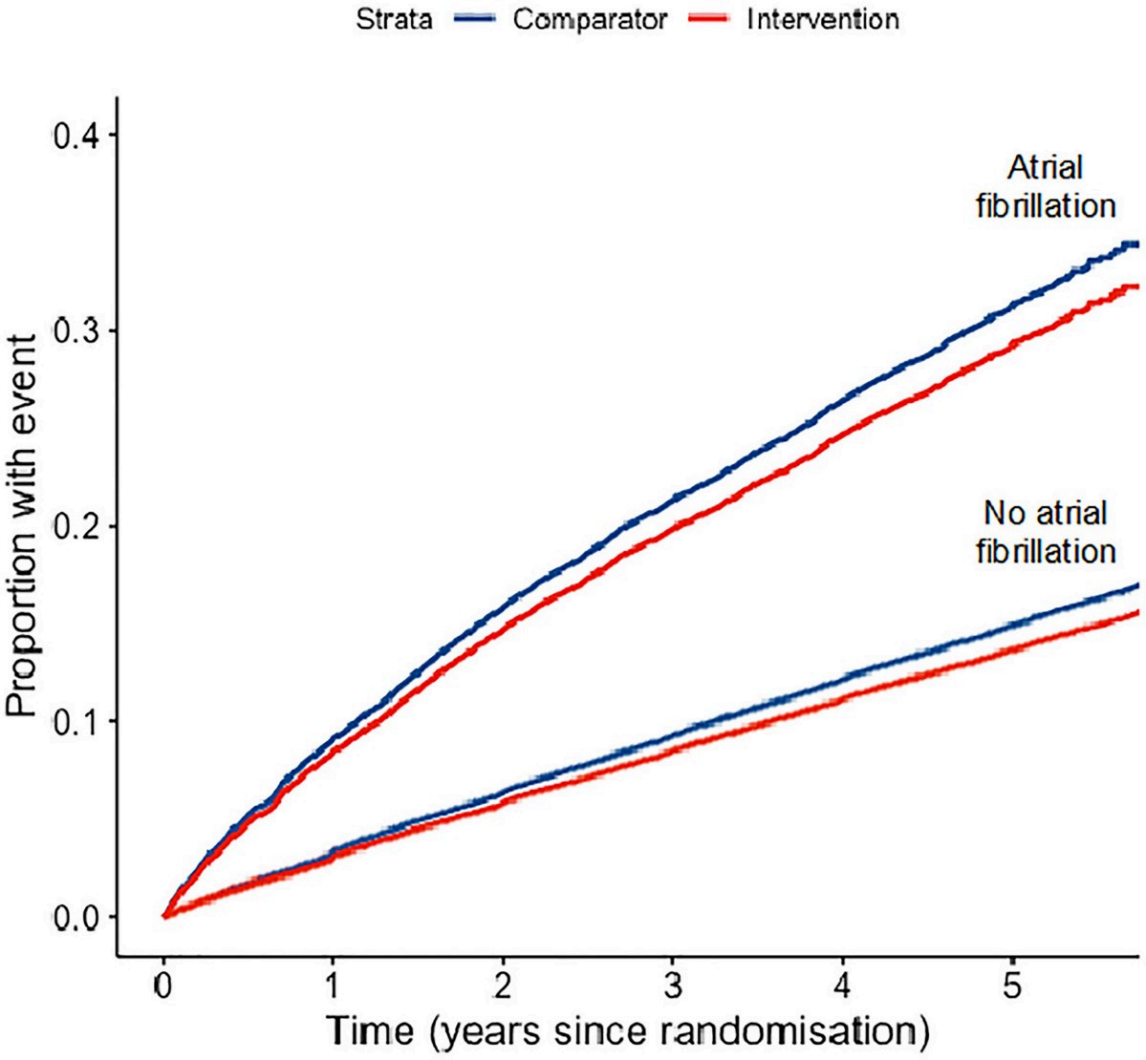
Cumulative event rates for the primary outcome (major cardiovascular events) by treatment arm, stratified by presence of atrial fibrillation at baseline. Shown are estimates of the proportions of patients with major cardiovascular events (primary composite endpoint) according to treatment arm (intervention versus comparator as defined in treatment comparisons in the methods) for patients with atrial fibrillation (top lines) and without atrial fibrillation at baseline (bottom lines). These curves were created for the overall population included in this study without accounting for stratification by trial.

**Fig 4:**
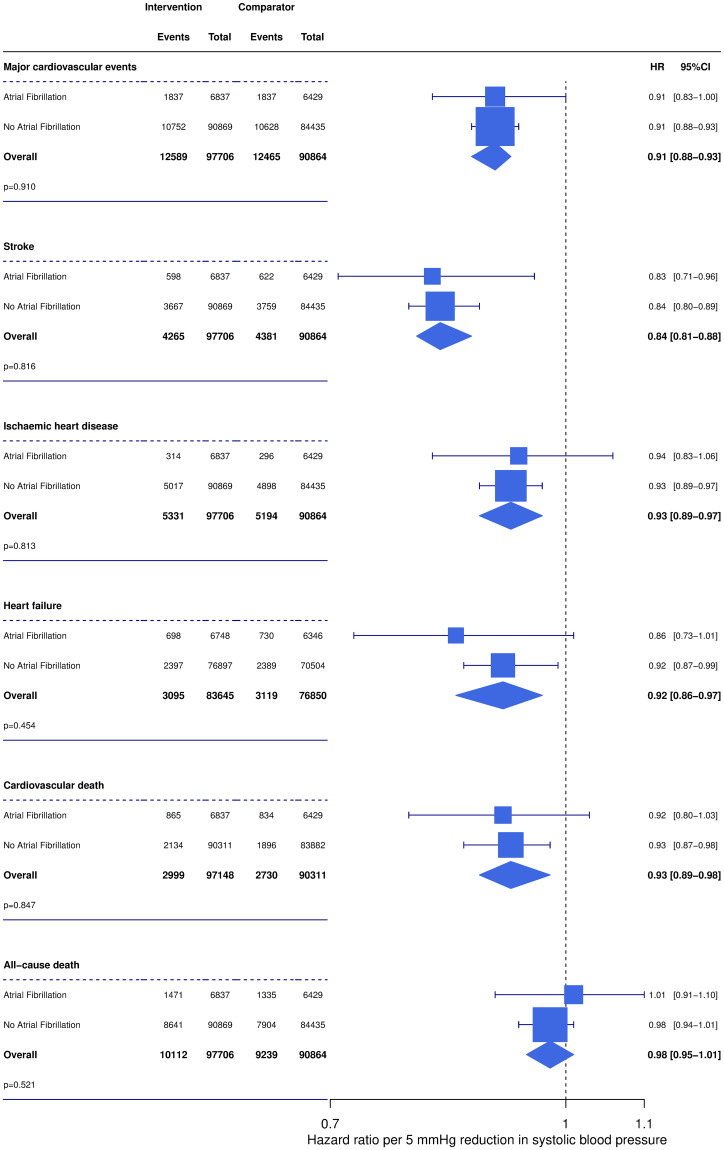
Effect of blood pressure lowering treatment on primary and secondary outcomes, stratified by presence of atrial fibrillation at baseline. Forest plot displays the HRs and 95% CIs for each outcome adjusted for a 5-mm Hg systolic blood pressure reduction. Further details on adjustment provided in the Methods. *P* values for test of difference between subgroups. CI, confidence interval; HR, hazard ratio.

Over a median follow up of 4.5 years (interquartile range 3.8 to 5.3), 3,674 (27.8%) and 21,380 (12.2%) patients with and without AF, respectively, developed a major cardiovascular event. This translates into a rate of major cardiovascular events of 73 and 28 per 1,000 patient-years for patients with and without AF, respectively (Fig 3). Each 5 mm Hg SBP lowering reduced the risk of major cardiovascular events by about 10% in patients with and without AF at baseline (HR 0.91, 95% confidence interval (CI) [0.83 to 1.00] versus HR 0.91, 95% CI [0.88 to 0.93] for patients with and without AF at baseline, respectively) (Figs 3 and 4). Furthermore, there was no evidence that the risk reduction for any of the primary and secondary outcomes achieved by BP-lowering treatment was different between patients with and without AF (Fig 4). Adjustment for the average reduction in SBP of 3.7 mm Hg was consistent with our main adjustment to 5 mm Hg SBP reduction and showed no difference between patients with and without AF (S2 Fig).

**Fig 5:**
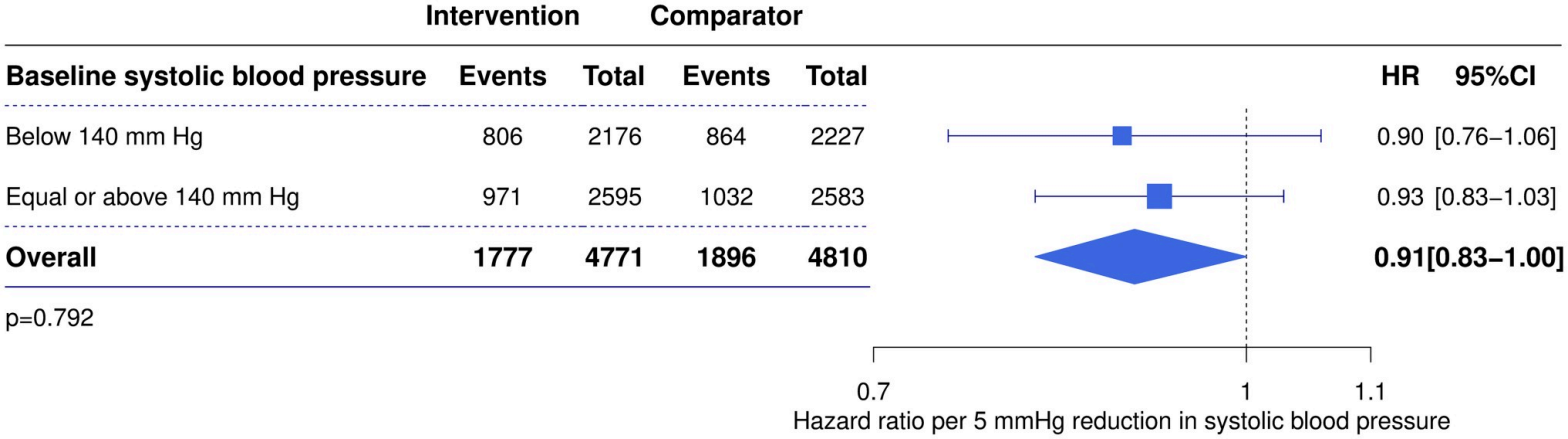
Effect of blood pressure-lowering treatment on major cardiovascular events stratified by baseline systolic blood pressure in patients with atrial fibrillation. Forest plot displays the HRs and 95% CIs for major cardiovascular events for a 5-mm Hg systolic blood pressure reduction in patients with atrial fibrillation with baseline systolic blood pressure below or above 140 mm Hg. *P* value for test of difference between subgroups. CI, confidence interval; HR, hazard ratio.

Subgroup analysis in patients with AF showed no evidence that the relative risk reduction in major cardiovascular events varied according to baseline SBP (test for linear trend *P* = 0.992). There was also no difference in treatment effects between patients with baseline SBP below and above 140 mmHg (*P* = 0.792) (Fig 5).

**Fig 6:**
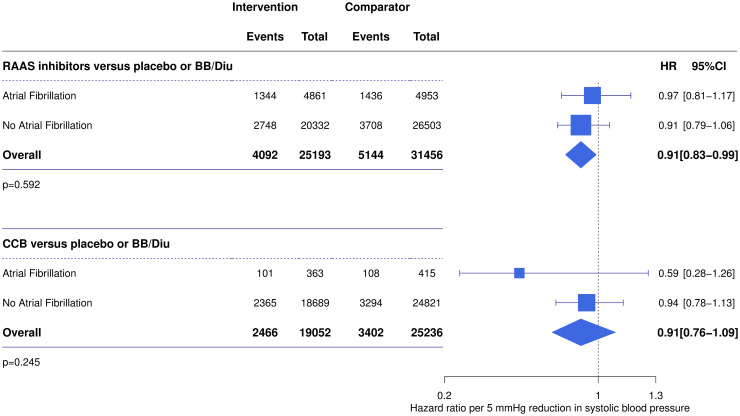
Effect of blood pressure-lowering treatment on major cardiovascular events stratified by drug class. Forest plot displays the HRs and 95% CIs for major cardiovascular events for a 5-mm Hg systolic blood pressure reduction for RAAS inhibitors-based and CCB-based regimens in comparison with placebo or BB with or without Diu. *P* values for test of difference between subgroups. BB, beta-blocker; CCB, calcium channel blocker; CI, confidence interval; Diu, diuretic; HR, hazard ratio; RAAS, renin-angiotensin-aldosterone system.

Six trials were included in the comparison of renin-angiotensin-aldosterone system (RAAS)-inhibitors versus placebo or standard treatment (i.e., beta-blocker and/or diuretic), including 56,649 participants. Four trials were included in the comparison of calcium channel blockers (CCB)-based regimens versus placebo or standard treatment (i.e., beta-blocker and/or diuretic), including 44,288 participants. There was no evidence of a difference in the effects of treatment regimens based on RAAS inhibitors or CCB between patients with and without AF at baseline (*P* = 0.245 and *P* = 0.909 for RAAS-based and CCB-based regimens, respectively) (Fig 6). However, the CIs were wide due to the relatively small number of AF participants.

Sensitivity analysis using only trials that contributed to both subgroups, that is the 14 trials that included both participants with and without AF at baseline and thus allowed estimation of the within-trial interaction between treatment and AF at baseline, showed broadly similar results to those from the main analyses. However, the smaller sample size meant that the CIs were wider, particularly in patients with AF at baseline (S7 Table). Sensitivity analysis using a two-stage approach yielded similar estimates to the one-stage approach overall and for subgroup analysis according to AF status at baseline (S8 Table). Several additional sensitivity analyses were requested by the reviewers which were conducted and broadly supported our main findings: We reran the models using an unadjusted approach for SBP and found no material change in our results (S9 Table). The results of fixed and random effects two-stage models were consistent and further supported the robustness of the main model (S8 Table). To rule out the effects of treatment in ACTIVE-I trial were driven by inclusion of patients with HF, we conducted a sensitivity analysis in which we excluded the patients with a diagnosis of HF at baseline in ACTIVE-I trial. As shown in S10 Table, results were consistent with our main analysis. Additional adjustment for baseline SBP, cardiovascular disease status, and diabetes at baseline had no impact on our main findings (S11 Table). Finally, no material change was also seen after excluding the trials with moderate risk of bias (S12 Table).

## Discussion

This study showed that BP-lowering treatment affords a similar relative risk reduction in major cardiovascular events in patients with and without AF, with no evidence that treatment effects differed between those subgroups for any of the primary and secondary outcomes. Overall, each 5-mm Hg reduction in SBP resulted in an approximately 10% lower risk of major cardiovascular events both in patients with and without AF at baseline. Furthermore, there was no evidence that in patients with AF the relative risk reduction varied according to baseline SBP [38].

Although absolute risks are better estimated from population-based observational studies, the almost 3-fold higher event rate that we observed in patients with AF at baseline compared with those without AF reflects their higher cardiovascular risk. This is in keeping with previous observational studies which reported that AF was associated with a 2- to 5-fold higher risk of major cardiovascular events in comparison with patients without AF [4,39,40]. Therefore, the same relative risk reduction afforded by BP-lowering treatment would most likely achieve a greater absolute risk reduction in patients with AF than in patients without AF at baseline.

It is thus a paradox that much of the focus of AF-related research has been on anticoagulation for stroke prevention, and strategies for rate-control or restoration of sinus rhythm, when the relative and absolute risk for cardiovascular events like HF and ischaemic heart disease is greater than that of stroke in patients with AF [40]. In addition, even with optimal anticoagulation and rate or rhythm control, the risk of stroke in patients with AF remains high (about 1.5% per year) [41] and it seems to result from associated risk factors rather than treatment failure [42,43]. Therefore, management of associated cardiovascular risk factors, among which high BP with an estimated prevalence of 70% is the most common, seems a priority to improve cardiovascular outcomes and survival in the high-risk group of patients with AF [44]. In this context, our study provides compelling evidence that pharmacological BP-lowering treatment is an effective strategy to prevent cardiovascular events overall as well as to address the residual risk of stroke.

Although the most recent AF guidelines issued by the European Society of Cardiology state that “good blood pressure control should form an integral part of the management of AF patients,” randomised evidence has been lacking to support those recommendations [7]. This uncertainty underpins the cautious AF guidelines of the American College of Cardiology, which despite mentioning that “appropriate control of risk factors like hypertension substantially reduces stroke risk,” make no specific recommendations about BP management in patient with AF [9]. Thus far, evidence on potential importance of BP reduction in patients with AF comes from observational studies, wherein the ideal SBP target for the prevention of cardiovascular events was found to be in the range of 120 to 129 mmHg [4]. Our study fills this gap in randomised evidence. We found that patients with AF included in trials had a relatively low baseline SBP, with almost half of them having a SBP of less than 140 mmHg prior to randomisation, commonly not recommended for treatment [38].

Hypertension guidelines recommend that drugs with shared rate- and BP-lowering properties (e.g., non-dihydropyridine CCBs and beta-blockers) should be preferred in patients with AF and high BP [38,45]. However, those recommendations are based on the indication of those drugs for rate control as thus far whether pharmacological BP lowering decreases cardiovascular risk in patients with AF has not been demonstrated. In addition, the lack of evidence for class-specific effects together with the linear association between the HR for major cardiovascular events and the intensity of SBP reduction showed in this study suggest that the intensity of BP lowering is more important than the specific drugs used to achieve it when it comes to prevention cardiovascular events. Therefore, until further research clarifies whether any drug class can achieve superior risk reduction for equivalent BP reduction, BP lowering irrespective of the drug class should be viewed as an effective strategy to decrease the high cardiovascular risk of patients with AF.

The main strength of this IPD meta-analysis is the analysis of the effects of BP-lowering treatment in a large number of patients with baseline AF included into RCTs, their comparison with patients without AF, the long follow-up time, and the more than 20,000 major cardiovascular events. The robustness of the main conclusions to sensitivity analyses and the consistency of the estimates provided by different methods further support the conclusions drawn. However, some limitations deserve to be acknowledged. First, there is a possibility of selection bias, as IPD was not obtainable for all the trials eligible for inclusion in the BPLTTC. While it remains uncertain whether those trials had collected data on AF status at baseline, it seems unlikely that lack of contribution to the BPLTTC has biased our estimates. This is supported by the consistency of the effects of BP reduction on cardiovascular outcomes in our set of studies with the estimates in a more comprehensive, albeit tabular meta-analysis (roughly 10% relative risk reduction per 5 mm Hg SBP reduction) [10]. Second, the number of participants with AF was modest considering the total number of participants in the BPLTTC dataset because only a fraction of the trials reported AF at baseline and the relatively low rate of AF in the trial populations. On the other hand, this means that even if a degree of misclassification is present due to omitted disclosure or the paroxysmal nature of the arrhythmia, a material impact on treatment effect estimates would be unlikely. Third, although it would have been interesting to compare the effects of other drug classes, particularly in head-to-head comparisons, only a small fraction of the trials included in the BPLTTC reported AF at baseline and were thus eligible for this study. Fourth, although concerns have been raised about the variability of BP measurement in patients with AF, which could have biased our estimates, there was no evidence of this in our population [46]. Finally, much of the weight of the analysis in the current report was driven by the ACTIVE-I trial, which included about 30% of patients with HF. However, excluding the HF patients from the analysis did not have a material impact on our findings of no interaction between AF status at baseline and the effect of BP lowering treatment.

In conclusion, this study demonstrated that BP-lowering treatment reduces the risk of major cardiovascular events in patients with AF to a similar extent to that of patients without AF, with no evidence that treatment effects varied according to baseline SBP or drug class. Owing to their higher absolute cardiovascular risk, treatment in patients with AF is likely to result in greater absolute risk reduction than in patients without AF. Clinical guidelines should be updated to recommend pharmacological BP lowering for prevention of cardiovascular events in patients with AF.

## Supporting information

S1 Fig Funnel plot for assessment of publication (acquisition) bias on the effect of blood pressure reduction and risk of major cardiovascular event. (DOCX)

S2 Fig Sensitivity analysis for the effect of blood pressure-lowering treatment on primary and secondary outcomes, stratified by presence of atrial fibrillation at baseline and adjusted for a 3.7-mm Hg systolic blood pressure reduction. (DOCX)

S1 Table Difference in systolic blood pressure reduction between arms for each trial. (DOCX)

S2 Table Assessment of risk of bias. (DOCX)

S3 Table Number of trials available for drug class comparisons. (DOCX)

S4 Table Treatment comparisons for subgroup analyses by drug class. (DOCX)

S5 Table Summary of included trials. (DOCX)

S6 Table Baseline characteristics of the participants included in atrial fibrillation meta-analyses stratified by trial. (DOCX)

S7 Table Sensitivity analyses including only trials that included patients with and without atrial fibrillation at baseline. (DOCX)

S8 Table Fixed and random effects two-stage meta-analyses. (DOCX)

S9 Table Unadjusted effect of blood pressure-lowering treatment on primary and secondary outcomes, stratified by the presence of atrial fibrillation at baseline. (DOCX)

S10 Table Sensitivity analysis for the effect of blood pressure-lowering treatment on primary and secondary outcomes, stratified by the presence of atrial fibrillation at baseline, excluding the patients with the diagnosis of heart failure at baseline in ACTIVE-I trial. (DOCX)

S11 Table Sensitivity analysis for the effect of blood pressure-lowering treatment on primary and secondary outcomes, stratified by the presence of atrial fibrillation at baseline, after adjustment for baseline systolic blood pressure, cardiovascular disease status, and diabetes status at baseline. (DOCX)

S12 Table Sensitivity analysis for the effect of blood pressure-lowering treatment on primary and secondary outcomes, stratified by the presence of atrial fibrillation at baseline, excluding the trials with moderate risk of bias. (DOCX)

S1 Methods (DOCX)

S1 PRISMA Checklist (DOCX)

S1 Protocol (DOCX)

## Abbreviations

AF: atrial fibrillation
BP: blood pressure
BPLTTC: Blood Pressure Lowering Treatment Trialists’ Collaboration
CCB: calcium channel blockers
CI: confidence interval
DBP: diastolic BP
HF: heart failure
HR: hazard ratio
IPD: individual participant data
PRISMA: Preferred Reporting Items for Systematic Reviews and Meta-Analyses
RAAS: renin-angiotensin-aldosterone system
RCT: randomised controlled trial
SBP: systolic BP

## References

[1] Chugh SS, Havmoeller R, Narayanan K, Singh D, Rienstra M, Benjamin EJ, et al. Worldwide epidemiology of atrial fibrillation: a Global Burden of Disease 2010 Study. Circulation. 2014;129(8):837–47. Epub 2013/12/19. 10.1161/CIRCULATIONAHA.113.005119 . 24345399 PMC4151302

[2] Lane DA, Skjoth F, Lip GYH, Larsen TB, Kotecha D. Temporal Trends in Incidence, Prevalence, and Mortality of Atrial Fibrillation in Primary Care. JAMA. 2017;6(5). Epub 2017/04/30. 10.1161/jaha.116.005155 . 28455344 PMC5524079

[3] Rahman F, Kwan GF, Benjamin EJ. Global epidemiology of atrial fibrillation. Nat Rev Cardiol. 2014;11(11):639–54. Epub 2014/08/13. 10.1038/nrcardio.2014.118 . 25113750

[4] Emdin CA, Anderson SG, Salimi-Khorshidi G, Woodward M, MacMahon S, Dwyer T, et al. Usual blood pressure, atrial fibrillation and vascular risk: evidence from 4.3 million adults. Int J Epidemiol. 2017;46(1):162–72. Epub 2016/05/05. 10.1093/ije/dyw053 . 27143136 PMC5407172

[5] Marijon E, Le Heuzey JY, Connolly S, Yang S, Pogue J, Brueckmann M, et al. Causes of death and influencing factors in patients with atrial fibrillation: a competing-risk analysis from the randomized evaluation of long-term anticoagulant therapy study. Circulation. 2013;128(20):2192–201. Epub 2013/09/11. 10.1161/CIRCULATIONAHA.112.000491 . 24016454

[6] Soliman EZ, Safford MM, Muntner P, Khodneva Y, Dawood FZ, Zakai NA, et al. Atrial fibrillation and the risk of myocardial infarction. JAMA Intern Med. 2014;174(1):107–14. Epub 2013/11/06. 10.1001/jamainternmed.2013.11912 . 24190540 PMC4115282

[7] Kirchhof P, Benussi S, Kotecha D, Ahlsson A, Atar D, Casadei B, et al. 2016 ESC Guidelines for the management of atrial fibrillation developed in collaboration with EACTS. Europace. 2016;18(11):1609–78. Epub 2016/11/04. 10.1093/europace/euw295 . 27567465

[8] Manolis AJ, Rosei EA, Coca A, Cifkova R, Erdine SE, Kjeldsen S, et al. Hypertension and atrial fibrillation: diagnostic approach, prevention and treatment. Position paper of the Working Group ’Hypertension Arrhythmias and Thrombosis’ of the European Society of Hypertension. J Hypertens. 2012;30(2):239–52. Epub 2011/12/22. 10.1097/HJH.0b013e32834f03bf . 22186358

[9] January CT, Wann LS, Alpert JS, Calkins H, Cigarroa JE, Cleveland JC Jr., et al. 2014 AHA/ACC/HRS guideline for the management of patients with atrial fibrillation: a report of the American College of Cardiology/American Heart Association Task Force on Practice Guidelines and the Heart Rhythm Society. JACC. 2014;64(21):e1–76. Epub 2014/04/02. 10.1016/j.jacc.2014.03.022 . 24685669

[10] Ettehad D, Emdin CA, Kiran A, Anderson SG, Callender T, Emberson J, et al. Blood pressure lowering for prevention of cardiovascular disease and death: a systematic review and meta-analysis. Lancet. 2016;387(10022):957–67. Epub 2016/01/03. 10.1016/S0140-6736(15)01225-8 . 26724178

[11] Wijesurendra RS, Casadei B. Mechanisms of atrial fibrillation. Heart. 2019;105 (24) 1860–7. 10.1136/heartjnl-2018-314267 31444267

[12] Yusuf S, Healey JS, Pogue J, Chrolavicius S, Flather M, Hart RG, et al. Irbesartan in patients with atrial fibrillation. NEJM. 2011;364(10):928–38. Epub 2011/03/11. 10.1056/NEJMoa1008816 . 21388310

[13] Rahimi K, Canoy D, Nazarzadeh M, Salimi-Khorshidi G, Woodward M, Teo K, et al. Investigating the stratified efficacy and safety of pharmacological blood pressure-lowering: an overall protocol for individual patient-level data meta-analyses of over 300 000 randomised participants in the new phase of the Blood Pressure Lowering Treatment Trialists’ Collaboration (BPLTTC). BMJ Open. 2019;9(5):e028698. Epub 2019/05/28. 10.1136/bmjopen-2018-028698 . 31123005 PMC6538087

[14] Sterne JAC, Savović J, Page MJ, Elbers RG, Blencowe NS, Boutron I, et al. RoB 2: a revised tool for assessing risk of bias in randomised trials. BMJ. 2019;366:l4898. Epub 2019/08/30. 10.1136/bmj.l4898 . 31462531

[15] Schmid CH, Stark PC, Berlin JA, Landais P, Lau J. Meta-regression detected associations between heterogeneous treatment effects and study-level, but not patient-level, factors. J Clin Epidemiol. 2004;57(7):683–97. Epub 2004/09/11. 10.1016/j.jclinepi.2003.12.001 . 15358396

[16] Simmonds MC, Higgins JP. Covariate heterogeneity in meta-analysis: criteria for deciding between meta-regression and individual patient data. Stat Med. 2007;26(15):2982–99. Epub 2007/01/02. 10.1002/sim.2768 . 17195960

[17] Austin PC, Lee DS, Fine JP. Introduction to the Analysis of Survival Data in the Presence of Competing Risks. Circulation. 2016;133(6):601–9. Epub 2016/02/10. 10.1161/CIRCULATIONAHA.115.017719 . 26858290 PMC4741409

[18] Dahlof B, Sever PS, Poulter NR, Wedel H, Beevers DG, Caulfield M, et al. Prevention of cardiovascular events with an antihypertensive regimen of amlodipine adding perindopril as required versus atenolol adding bendroflumethiazide as required, in the Anglo-Scandinavian Cardiac Outcomes Trial-Blood Pressure Lowering Arm (ASCOT-BPLA): a multicentre randomised controlled trial. Lancet. 2005;366(9489):895–906. Epub 2005/09/13. 10.1016/S0140-6736(05)67185-1 . 16154016

[19] Hansson L, Lindholm LH, Niskanen L, Lanke J, Hedner T, Niklason A, et al. Effect of angiotensin-converting-enzyme inhibition compared with conventional therapy on cardiovascular morbidity and mortality in hypertension: the Captopril Prevention Project (CAPPP) randomised trial. Lancet. 1999;353(9153):611–6. Epub 1999/02/25. 10.1016/s0140-6736(98)05012-0 . 10030325

[20] Ogihara T, Saruta T, Rakugi H, Saito I, Shimamoto K, Matsuoka H, et al. Combinations of olmesartan and a calcium channel blocker or a diuretic in elderly hypertensive patients: a randomized, controlled trial. J Hypertens. 2014;32(10):2054–63; discussiom 63. Epub 2014/07/08. 10.1097/HJH.0000000000000281 . 24999799 PMC4166009

[21] Matsuzaki M, Ogihara T, Umemoto S, Rakugi H, Matsuoka H, Shimada K, et al. Prevention of cardiovascular events with calcium channel blocker-based combination therapies in patients with hypertension: a randomized controlled trial. J Hypertens. 2011;29(8):1649–59. Epub 2011/05/26. 10.1097/HJH.0b013e328348345d . 21610513

[22] Kasanuki H, Hagiwara N, Hosoda S, Sumiyoshi T, Honda T, Haze K, et al. Angiotensin II receptor blocker-based vs. non-angiotensin II receptor blocker-based therapy in patients with angiographically documented coronary artery disease and hypertension: the Heart Institute of Japan Candesartan Randomized Trial for Evaluation in Coronary Artery Disease (HIJ-CREATE). Eur Heart J. 2009;30(10):1203–12. 10.1093/eurheartj/ehp101 . 19346521

[23] Hansson L, Hedner T, Lund-Johansen P, Kjeldsen SE, Lindholm LH, Syvertsen JO, et al. Randomised trial of effects of calcium antagonists compared with diuretics and beta-blockers on cardiovascular morbidity and mortality in hypertension: the Nordic Diltiazem (NORDIL) study. Lancet. 2000;356(9227):359–65. Epub 2000/09/06. 10.1016/s0140-6736(00)02526-5 . 10972367

[24] Hansson L, Lindholm LH, Ekbom T, Dahlof B, Lanke J, Schersten B, et al. Randomised trial of old and new antihypertensive drugs in elderly patients: cardiovascular mortality and morbidity the Swedish Trial in Old Patients with Hypertension-2 study. Lancet. 1999;354(9192):1751–6. Epub 1999/11/30. 10.1016/s0140-6736(99)10327-1 . 10577635

[25] Julius S, Kjeldsen SE, Weber M, Brunner HR, Ekman S, Hansson L, et al. Outcomes in hypertensive patients at high cardiovascular risk treated with regimens based on valsartan or amlodipine: the VALUE randomised trial. Lancet. 2004;363(9426):2022–31. Epub 2004/06/23. 10.1016/S0140-6736(04)16451-9 . 15207952

[26] Yusuf S, Teo KK, Pogue J, Dyal L, Copland I, Schumacher H, et al. Telmisartan, ramipril, or both in patients at high risk for vascular events. NEJM. 2008;358(15):1547–59. Epub 2008/04/02. 10.1056/NEJMoa0801317 . 18378520

[27] Patel A, MacMahon S, Chalmers J, Neal B, Woodward M, Billot L, et al. Effects of a fixed combination of perindopril and indapamide on macrovascular and microvascular outcomes in patients with type 2 diabetes mellitus (the ADVANCE trial): a randomised controlled trial. Lancet. 2007;370(9590):829–40. Epub 2007/09/04. 10.1016/S0140-6736(07)61303-8 . 17765963

[28] Amery A, Birkenhager W, Brixko P, Bulpitt C, Clement D, Deruyttere M, et al. Mortality and morbidity results from the European Working Party on High Blood Pressure in the Elderly trial. Lancet. 1985;1(8442):1349–54. Epub 1985/06/15. 10.1016/s0140-6736(85)91783-0 . 2861311

[29] Staessen JA, Fagard R, Thijs L, Celis H, Arabidze GG, Birkenhager WH, et al. Randomised double-blind comparison of placebo and active treatment for older patients with isolated systolic hypertension. The Systolic Hypertension in Europe (Syst-Eur) Trial Investigators. Lancet. 1997;350(9080):757–64. Epub 1997/09/23. 10.1016/s0140-6736(97)05381-6 . 9297994

[30] Yusuf S, Teo K, Anderson C, Pogue J, Dyal L, Copland I, et al. Effects of the angiotensin-receptor blocker telmisartan on cardiovascular events in high-risk patients intolerant to angiotensin-converting enzyme inhibitors: a randomised controlled trial. Lancet. 2008;372(9644):1174–83. Epub 2008/09/02. 10.1016/S0140-6736(08)61242-8 . 18757085

[31] Cushman WC, Evans GW, Byington RP, Goff DC Jr., Grimm RH Jr., Cutler JA, et al. Effects of intensive blood-pressure control in type 2 diabetes mellitus. NEJM. 2010;362(17):1575–85. Epub 2010/03/17. 10.1056/NEJMoa1001286 . 20228401 PMC4123215

[32] PROGRESS Collaborative Group. Randomised trial of a perindopril-based blood-pressure-lowering regimen among 6,105 individuals with previous stroke or transient ischaemic attack. Lancet. 2001;358(9287):1033–41. Epub 2001/10/09. 10.1016/S0140-6736(01)06178-5 . 11589932

[33] ALLHAT Collaborative Research Group. Major outcomes in high-risk hypertensive patients randomized to angiotensin-converting enzyme inhibitor or calcium channel blocker vs diuretic: The Antihypertensive and Lipid-Lowering Treatment to Prevent Heart Attack Trial (ALLHAT). JAMA. 2002;288(23):2981–97. Epub 2002/12/20. 10.1001/jama.288.23.2981 . 12479763

[34] Ogihara T, Saruta T, Rakugi H, Saito I, Shimamoto K, Matsuoka H, et al. Combinations of olmesartan and a calcium channel blocker or a diuretic in elderly hypertensive patients: a randomized, controlled trial. J Hypertens. 2014;32(10):2054–63. Epub 2014/07/08. 10.1097/HJH.0000000000000281 . 24999799 PMC4166009

[35] Verdecchia P, Staessen JA, Angeli F, de Simone G, Achilli A, Ganau A, et al. Usual versus tight control of systolic blood pressure in non-diabetic patients with hypertension (Cardio-Sis): an open-label randomised trial. Lancet. 2009;374(9689):525–33. Epub 2009/08/18. 10.1016/S0140-6736(09)61340-4 . 19683638

[36] Yui Y, Sumiyoshi T, Kodama K, Hirayama A, Nonogi H, Kanmatsuse K, et al. Comparison of nifedipine retard with angiotensin converting enzyme inhibitors in Japanese hypertensive patients with coronary artery disease: the Japan Multicenter Investigation for Cardiovascular Diseases-B (JMIC-B) randomized trial. Hypertens Res. 2004;27(3):181–91. Epub 2004/04/15. 10.1291/hypres.27.181 . 15080377

[37] Ogihara T, Nakao K, Fukui T, Fukiyama K, Ueshima K, Oba K, et al. Effects of candesartan compared with amlodipine in hypertensive patients with high cardiovascular risks: candesartan antihypertensive survival evaluation in Japan trial. Hypertension. 2008;51(2):393–8. Epub 2008/01/04. 10.1161/HYPERTENSIONAHA.107.098475 . 18172059

[38] Williams B, Mancia G, Spiering W, Agabiti Rosei E, Azizi M, Burnier M, et al. 2018 ESC/ESH Guidelines for the management of arterial hypertension. Eur Heart J. 2018;39(33):3021–104. 10.1093/eurheartj/ehy339 30165516

[39] Bansal N, Xie D, Sha D, Appel LJ, Deo R, Feldman HI, et al. Cardiovascular Events after New-Onset Atrial Fibrillation in Adults with CKD: Results from the Chronic Renal Insufficiency Cohort (CRIC) Study. JASN. 2018;29(12):2859–69. Epub 2018/11/01. 10.1681/ASN.2018050514 . 30377231 PMC6287862

[40] Odutayo A, Wong CX, Hsiao AJ, Hopewell S, Altman DG, Emdin CA. Atrial fibrillation and risks of cardiovascular disease, renal disease, and death: systematic review and meta-analysis. BMJ 2016;354:i4482. Epub 2016/09/08. 10.1136/bmj.i4482 . 27599725

[41] Ruff CT, Giugliano RP, Braunwald E, Hoffman EB, Deenadayalu N, Ezekowitz MD, et al. Comparison of the efficacy and safety of new oral anticoagulants with warfarin in patients with atrial fibrillation: a meta-analysis of randomised trials. Lancet. 2014;383(9921):955–62. Epub 2013/12/10. 10.1016/S0140-6736(13)62343-0 . 24315724

[42] Senoo K, Lip GY, Lane DA, Buller HR, Kotecha D. Residual Risk of Stroke and Death in Anticoagulated Patients According to the Type of Atrial Fibrillation: AMADEUS Trial. Stroke. 2015;46(9):2523–8. Epub 2015/07/25. 10.1161/STROKEAHA.115.009487 . 26205373

[43] Freedman B, Martinez C, Katholing A, Rietbrock S. Residual Risk of Stroke and Death in Anticoagulant-Treated Patients With Atrial Fibrillation. JAMA Cardiol. 2016;1(3):366–8. Epub 2016/07/22. 10.1001/jamacardio.2016.0393 . 27438123

[44] Lip GY, Laroche C, Dan GA, Santini M, Kalarus Z, Rasmussen LH, et al. A prospective survey in European Society of Cardiology member countries of atrial fibrillation management: baseline results of EURObservational Research Programme Atrial Fibrillation (EORP-AF) Pilot General Registry. Europace. 2014;16(3):308–19. Epub 2013/12/20. 10.1093/europace/eut373 . 24351881

[45] Whelton PK, Carey RM, Aronow WS, Casey DE Jr., Collins KJ, Dennison Himmelfarb C, et al. 2017 ACC/AHA/AAPA/ABC/ACPM/AGS/APhA/ASH/ASPC/NMA/PCNA Guideline for the Prevention, Detection, Evaluation, and Management of High Blood Pressure in Adults: A Report of the American College of Cardiology/American Heart Association Task Force on Clinical Practice Guidelines. Hypertension. 2018;71(6):e13–e115. Epub 2017/11/15. 10.1161/HYP.0000000000000065 . 29133356

[46] Stergiou GS, Kollias A, Destounis A, Tzamouranis D. Automated blood pressure measurement in atrial fibrillation: a systematic review and meta-analysis. J Hypertens. 2012;30(11):2074–82. Epub 2012/08/24. 10.1097/HJH.0b013e32835850d7 . 22914573

