# Supplementary material for: Blood pressure-lowering treatment for the prevention of cardiovascular events in patients with atrial fibrillation: An individual participant data meta-analysis
Source: PLoS Med. 2021 Jun 1;18(6):e1003599. doi: 10.1371/journal.pmed.1003599 (PMC8168843; doi:10.1371/journal.pmed.1003599)
Supplement: S1 Fig — (DOCX) [file pmed.1003599.s001.docx]

### S1 Fig. Funnel plot for assessment of publication (acquisition) bias on the effect of blood pressure reduction and risk of major cardiovascular event.


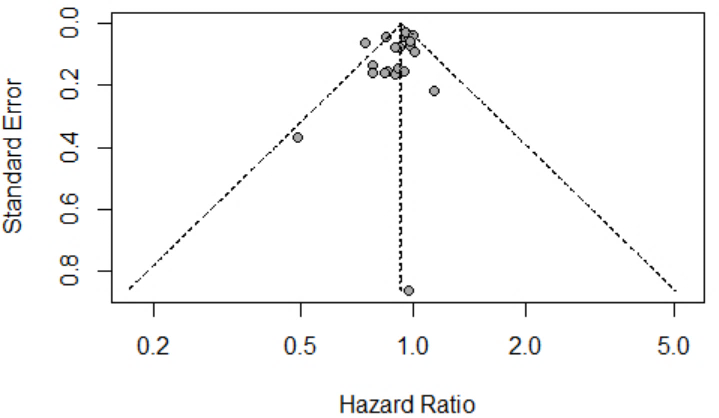


There was no evidence of publication bias according to the funnel plot and Egger’s regression test (T statistics = -1.43, df = 20, p-value = 0.1675, bias coefficient -0.68, standard error 0.48).
