# Supplementary material for: Blood pressure-lowering treatment for the prevention of cardiovascular events in patients with atrial fibrillation: An individual participant data meta-analysis
Source: PLoS Med. 2021 Jun 1;18(6):e1003599. doi: 10.1371/journal.pmed.1003599 (PMC8168843; doi:10.1371/journal.pmed.1003599)
Supplement: S2 Fig — (DOCX) [file pmed.1003599.s002.docx]

### S2 Fig. Sensitivity analysis for the effect of blood pressure-lowering treatment on primary and secondary outcomes, stratified by presence of atrial fibrillation at baseline and adjusted for a 3.7-mmHg systolic blood pressure reduction.

Forest plot displays the hazard ratios (HR) and 95% confidence intervals (CI) for each outcome. P-values: test of difference between subgroups.
