# Supplementary material for: Blood pressure-lowering treatment for the prevention of cardiovascular events in patients with atrial fibrillation: An individual participant data meta-analysis
Source: PLoS Med. 2021 Jun 1;18(6):e1003599. doi: 10.1371/journal.pmed.1003599 (PMC8168843; doi:10.1371/journal.pmed.1003599)
Supplement: S1 Table — (DOCX) [file pmed.1003599.s003.docx]

### S1 Table: Difference in systolic blood pressure reduction between arms for each trial

| Trial | Intervention | Comparator | SBP reduction between trial arms (mmHg) |
| --- | --- | --- | --- |
| ACCORD | More intense treatment | Less intense treatment | 12.4 |
| ACTIVE-I | ARB | Placebo | 2.6 |
| ADVANCE | ACEI and Diuretic | Placebo | 6.7 |
| ALLHAT | Diuretic | ACEI or CCB | 2.2 |
| ASCOT | CCB and ACEI | BB and Diuretic | 3.9 |
| CAPPP | BB and Diuretic | ACEI | 1.3 |
| CARDIO-SIS | More intense treatment | Less intense treatment | 4.7 |
| CASE-J | ARB | CCB | 2.3 |
| COLM | ARB and CCB | ARB and Diuretic | 0.3 |
| COPE | CCB and ARB | CCB and Diuretic or CCB and BB | 0.3 |
| DUTCH-TIA | BB | Placebo | 4.4 |
| EWPHE | Diuretic | Placebo | 21.3 |
| HIJCREATE | ARB | BB and Diuretic | 1.0 |
| JMICB | ACEI | CCB | 3.8 |
| NORDIL | BB and Diuretic | CCB | 3.1 |
| ONTARGET | ARB and ACEI | ACEI or ARB | 2.3 |
| PROGRESS | ACEI and Diuretic | Placebo | 8.2 |
| SHEP | BB and Diuretic | Placebo | 14.1 |
| STOP-2 | ACEI or CCB | BB and Diuretic | 2.3 |
| SYSTEUR | CCB | Placebo | 9.4 |
| TRANSCEND | ARB | Placebo | 4.7 |
| VALUE | CCB | ARB | 2.4 |

ACEI, angiotensin-converting enzyme inhibitor; ARB, angiotensin receptor blocker; BB, beta-blocker; CCB, calcium channel blocker; SBP, systolic blood pressure reduction
