# Supplementary material for: Blood pressure-lowering treatment for the prevention of cardiovascular events in patients with atrial fibrillation: An individual participant data meta-analysis
Source: PLoS Med. 2021 Jun 1;18(6):e1003599. doi: 10.1371/journal.pmed.1003599 (PMC8168843; doi:10.1371/journal.pmed.1003599)
Supplement: S2 Table — (DOCX) [file pmed.1003599.s004.docx]

### S2 Table. Assessment of risk of bias

| Trial | Randomization process | Deviations from intended interventions | Missing outcome data | Measurement of the outcome | Selection of the reported result | Overall |
| --- | --- | --- | --- | --- | --- | --- |
| ACCORD | Low | Low | Low | Low | Low | Low |
| ACTIVE-I | Low | Low | Low | Low | Low | Low |
| ADVANCE | Low | Low | Low | Low | Low | Low |
| ALLHAT | Low | Low | Low | Low | Low | Low |
| ASCOT | Low | Low | Low | Low | Low | Low |
| CAPPP | Moderate | Low | Low | Low | Low | Moderate |
| CARDIO-SIS | Low | Low | Low | Low | Low | Low |
| CASE-J | Low | Low | Low | Low | Low | Low |
| COLM | Low | Low | Low | Low | Low | Low |
| COPE | Low | Low | Low | Low | Low | Low |
| DUTCH-TIA | Low | Low | Low | Low | Low | Low |
| EWPHE | Low | Moderate | Low | Low | Low | Moderate |
| HIJCREATE | Low | Low | Low | Low | Low | Low |
| JMICB | Low | Low | Low | Low | Low | Low |
| NORDIL | Low | Low | Low | Low | Low | Low |
| ONTARGET | Low | Low | Low | Low | Low | Low |
| PROGRESS | Low | Low | Low | Low | Low | Low |
| SHEP | Low | Low | Low | Low | Low | Low |
| STOP-2 | Low | Low | Low | Low | Low | Low |
| SYSTEUR | Low | Low | Low | Low | Low | Low |
| TRANSCEND | Low | Low | Low | Low | Low | Low |
| VALUE | Low | Low | Low | Low | Low | Low |
