# Supplementary material for: Blood pressure-lowering treatment for the prevention of cardiovascular events in patients with atrial fibrillation: An individual participant data meta-analysis
Source: PLoS Med. 2021 Jun 1;18(6):e1003599. doi: 10.1371/journal.pmed.1003599 (PMC8168843; doi:10.1371/journal.pmed.1003599)
Supplement: S3 Table — (DOCX) [file pmed.1003599.s005.docx]

### S3 Table. Number of trials available for drug class comparisons

| Drug classes |  | Number trials |
| --- | --- | --- |
| ARB+CCB | ARB+Diuretic | 1 |
| CCB+ACEI | BB+ Diuretic | 1 |
| ACEI/ARB | BB+ Diuretic | 3 |
| CCB | BB+ Diuretic | 2 |
| ACEI/ARB | CCB | 4 (only 2 trials with patients with AF) |
| ARB+CCB | CCB+BB | 1 |
| ARB+CCB | CCB+ Diuretic | 1 |
| ACEI | Diuretic | 1 |
| CCB | Diuretic | 1 |
| ACEI/ARB | Placebo | 2 |
| ACEI+ Diuretic | Placebo | 2 |
| Diu | Placebo | 1 |
| BB+ Diuretic | Placebo | 1 |
| CCB | Placebo | 1 |
| BB | Placebo | 1 |

ACEI, angiotensin-converting enzyme inhibitor; ARB, angiotensin receptor blocker; BB, beta-blocker; CCB, calcium channel blocker
