# Supplementary material for: Blood pressure-lowering treatment for the prevention of cardiovascular events in patients with atrial fibrillation: An individual participant data meta-analysis
Source: PLoS Med. 2021 Jun 1;18(6):e1003599. doi: 10.1371/journal.pmed.1003599 (PMC8168843; doi:10.1371/journal.pmed.1003599)
Supplement: S4 Table — (DOCX) [file pmed.1003599.s006.docx]

### S4 Table. Treatment comparisons for subgroup analyses by drug class

| Trial | Intervention | Comparator |
| --- | --- | --- |
| Renin-angiotensin-aldosterone system inhibitors | | |
| ACTIVE-I | ARB | Placebo |
| ALLHAT | ACEI | Diuretic |
| CAPPP | ACEI | BB and Diuretic |
| HIJCREATE | ARB | BB and Diuretic |
| STOP-2 | ACEI | BB and Diuretic |
| TRANSCEND | ARB | Placebo |
| Calcium channel blockers | | |
| ALLHAT | CCB | Diuretic |
| NORDIL | CCB | BB and Diuretic |
| STOP-2 | CCB | BB and Diuretic |
| SYSTEUR | CCB | Placebo |
| Calcium channel blockers vs RAAS inhibitors | | |
| ALLHAT | CCB | ACEI |
| CASE-J | CCB | ARB |
| JMICB | CCB | ACEI |
| VALUE | CCB | ARB |

ACEI, angiotensin-converting enzyme inhibitor; ARB, angiotensin receptor blocker; BB, beta-blocker; CCB, calcium channel blocker
