# Supplementary material for: Blood pressure-lowering treatment for the prevention of cardiovascular events in patients with atrial fibrillation: An individual participant data meta-analysis
Source: PLoS Med. 2021 Jun 1;18(6):e1003599. doi: 10.1371/journal.pmed.1003599 (PMC8168843; doi:10.1371/journal.pmed.1003599)
Supplement: S5 Table — (DOCX) [file pmed.1003599.s007.docx]

### S5 Table. Summary of included trials

**Trials comparing different drug classes**

| Study | Country | Inclusion criteria | Randomisation period | Randomisation groups | Intervention | N participants (% women) | Mean follow-up duration, years (SD) |
| --- | --- | --- | --- | --- | --- | --- | --- |
|  |  |  |  |  |  |  |  |
| ALLHAT | Multi-country (US, Canada, Puerto Rico, US Virgin Islands) | ≥55yrs, stage 1 or 2 hypertension + ≥1 CVD risk factor | Feb 1994 - Jan 1998 | All |  | 42418 (47) | 4.8 (1.4) |
|  |  |  |  | DIURET (Chlorthalidone) | >140/90 mmHg | 15255 (47) | 5 (1.4) |
|  |  |  |  | CCB (Amlodipine) | >140/90 mmHg | 9048 (47) | 5.1 (1.3) |
|  |  |  |  | ACE (Lisinopril) | >140/90 mmHg | 9054 (46) | 5 (1.4) |
|  |  |  |  | ALPHA (Doxazosin) | >140/90 mmHg | 9061 (46) | 4.1 (1.5) |
| ASCOT-BPLA | Multi-country (Denmark, Iceland, Finland, Norway, Sweden, UK, Ireland) | 40-79yrs, hypertension, ≥3 CV risk factors | Feb 1998 - May 2000 | All |  | 19257 (23) | 5.3 (1.4) |
|  |  |  |  | CCB (Amlodipine) | <140/90 mmHg w/DM or <130/80 mmHg w/o DM | 9639 (23) | 5.3 (1.3) |
|  |  |  |  | BETA (Atenolol) | <140/90 mmHg w/DM or <130/80 mmHg w/o DM | 9618 (23) | 5.2 (1.4) |
| CAPPP | Multi-country (Sweden/ Finland) | 25-66yrs, treated or untreated hypertension | Dec 1989 - Apr 1995 | All |  | 10985 (47) | 5.8 (1.4) |
|  |  |  |  | ACE (Captopril) | 90 mmHg DBP | 5492 (45) | 5.8 (1.4) |
|  |  |  |  | BETA/DIURET (Atenolol, Metoprolol/ HCTZ, bendrofluazide) | 90 mmHg DBP | 5493 (48) | 5.8 (1.4) |
| COLM | Japan | 65-84yrs, hypertension, history of CVD/CVD risk factors | Jul 2007 - Sep 2008 | All |  | 5141 (48) | 3 (0.9) |
|  |  |  |  | ARB/CCB (Olmesartan/ CCB) | <140/<90 mmHg | 2568 (48) | 3.1 (0.9) |
|  |  |  |  | ARB/DIURET (Olmesartan/ DIU) | <140/<90 mmHg | 2573 (48) | 3 (1) |
| COPE | Japan | 40-85yrs, ≥140mmHg SBP and/or ≥90mmHg DBP | Jun 2003 - Nov 2006 | All |  | 3293 (49) | 3.6 (1.1) |
|  |  |  |  | CCB/ARB (benidipine/ ARB) | <140/<90 mmHg | 1110 (49) | 3.6 (1) |
|  |  |  |  | CCB/BETA (benidipine/ BETA) | <140/<90 mmHg | 1089 (49) | 3.6 (1.1) |
|  |  |  |  | CCB/DIURET (benidipine/ thiazide) | <140/<90 mmHg | 1094 (49) | 3.6 (1.1) |
| HIJCREATE | Japan | Hospitalisation for coronary artery disease and hypertension, 20-80yrs | Jun 2001 - Apr 2004 | All | <130/<85 mmHg | 2049 (20) | 4 (1) |
|  |  |  |  | ARB (Candesartan) | <130/<85 mmHg | 1024 (18) | 4 (1) |
|  |  |  |  | Non-ARB therapy (including ACEIs) | <130/<85 mmHg | 1025 (21) | 4 (1) |
| NORDIL | Multi-country (Norway/ Sweden) | Hypertension, 50-74yrs, previously untreated | Oct 1992 - Dec 1996 | All |  | 10881 (51) | 4.2 (1.3) |
|  |  |  |  | CCB (Diltiazem) | DBP <90 mmHg | 5410 (51) | 4.2 (1.3) |
|  |  |  |  | BETA/DIURET (BB/ Thiazide diuretic) | DBP <90 mmHg | 5471 (51) | 4.3 (1.3) |
| ONTARGET | Multi-country | Coronary, peripheral or cerebrovascular disease or diabetes with end-organ damage | Jan 2002 - Aug 2003 | All |  | 25620 (27) | 4.8 (0.4) |
|  |  |  |  | ACE (Ramipril) | 10mg | 8576 (27) | 4.8 (0.4) |
|  |  |  |  | ARB (Telmisartan) | 80mg | 8542 (26) | 4.8 (0.4) |
|  |  |  |  | ACE/ARB (Ramipril/ Telmisartan) | 80mg/10mg | 8502 (26) | 4.8 (0.4) |
| STOP2 | Sweden | Hypertension, 70-84yrs | Sep 1992 - Dec 1994 | All |  | 6614 (67) | 4.5 (1.2) |
|  |  |  |  | BETA/DIURETICS (Atenolol/ metoprolol/ pindolol/ hydrocholo-thiazide + amiloride) | <160/95 mmHg | 2213 (68) | 4.6 (1.2) |
|  |  |  |  | ACE (Enalapril/ lisinopril) | <160/95 mmHg | 2205 (66) | 4.5 (1.2) |
|  |  |  |  | CCB (Felodipine/ isradipine) | <160/95 mmHg | 2196 (66) | 4.5 (1.2) |
| VALUE | Multi-country | ≥50yrs, hypertension, CVD risk factors, CVD | Sept 1997 - Dec 1999 | All |  | 15245 (42) | 4.2 (1.2) |
|  |  |  |  | ARB (Valsartan) | <140/90 mmHg | 7649 (42) | 4.2 (1.2) |
|  |  |  |  | CCB (Amlodipine) | <140/90 mmHg | 7596 (42) | 4.2 (1.2) |
| CASEJ | Japan | 20-85yrs, hypertension | Sep 2001 - Jan 2003 | All |  | 4703 (45) | 3.1 (0.8) |
|  |  |  |  | ARB (Candesartan) | <160/<90 mmHg (SBP target adjusted for age; <130/<85 mmHg in under-60s) | 2354 (46) | 3.1 (0.8) |
|  |  |  |  | CCB (Amlodipine) | <160/<90 mmHg (SBP target adjusted for age; <130/<85 mmHg in under-60s) | 2349 (43) | 3.1 (0.8) |
| JMIC-B | Japan | <75yrs, hypertension | Apr 1991 - May 1998 | All |  | 1650 (31) | 2.3 (1.1) |
|  |  |  |  | CCB (Nifedipine) | <150/<90 mmHg | 828 (32) | 2.4 (1) |
|  |  |  |  | ACE (Enalapril/ Imidapril/ Lisinopril) | <150/<90 mmHg | 822 (30) | 2.2 (1.1) |

**Placebo-controlled trials**

| Study | Country | Inclusion criteria | Randomisation period | Randomisation group | Intervention | N participants (% women) | Mean follow-up duration, years (SD) |
| --- | --- | --- | --- | --- | --- | --- | --- |
| ACTIVE | Multi-country | Permanent AF or ≥2 episodes of intermittent AF in past 6 months, ≥1 risk factor | Jun 2003 - May 2006 | All |  | 9016 (39) | 4.1 (1.2) |
|  |  |  |  | Treatment group | ARB (Irbesartan) | 4518 (39) | 4.1 (1.2) |
|  |  |  |  | Control group | Placebo | 4498 (39) | 4.1 (1.3) |
| ADVANCE | Multi-country | Type 2 diabetes (diagnosed aged ≥30yrs), ≥55yrs, history of CVD or CVD risk factors | Jul 2001 - Mar 2003 | All |  | 11140 (43) | 4.2 (0.9) |
|  |  |  |  | Treatment group | ACE/DIURET (Perindopril/Indapamide) | 5569 (42) | 4.2 (0.9) |
|  |  |  |  | Control group | Placebo | 5571 (43) | 4.2 (0.9) |
| EWPHE | Multi-country | ≥60yrs, hypertension (160-239/90-119 mmHg) |  | All |  | 840 (70) | 4.6 (2.9) |
|  |  |  |  | Treatment group | DIURET (Hydrochlorothiazide/triamterene) | 416 (69) | 4.7 (2.8) |
|  |  |  |  | Control group | Placebo | 424 (71) | 4.6 (3) |
| PROGRESS | Multi-country | History of stroke or TIA within previous 5 years | Jun 1995 - Nov 1997 | All |  | 6105 (30) | 3.9 (0.8) |
|  |  |  |  | Treatment group | ACE/DIURET (Perindopril/Indapamide) | 3051 (30) | 3.9 (0.8) |
|  |  |  |  | Control group | Placebo | 3054 (30) | 3.9 (0.8) |
| SHEP | USA | ≥60yrs, isolated systolic hypertension (160-219/<90 mmHg not on treatment) | Mar 1985 - Jan 1988 | All |  | 4714 (57) | 5.0 |
|  |  |  |  | Treatment group | BETA/DIURET (Chlorthalidone/Atenolol) | 2353 (56) | 5.0 |
|  |  |  |  | Control group | Placebo | 2361 (57) | 5.0 |
| SYSTEUR | Multi-country | ≥60yrs, 160-219/<95 mmHg | Dec 1988 - Jan 1997 | All |  | 4695 (67) | 2.6 (1.9) |
|  |  |  |  | Treatment group | CCB (Nitrendipine) | 2398 (67) | 2.6 (1.9) |
|  |  |  |  | Control group | Placebo | 2297 (66) | 2.6 (1.9) |
| TRANSCEND | Multi-country | Coronary, peripheral or cerebrovascular disease or diabetes with end-organ damage | Dec 2001 - May 2004 | All |  | 5926 (43) | 4.9 (0.6) |
|  |  |  |  | Treatment group | ARB (Telmisartan) | 2954 (43) | 4.9 (0.6) |
|  |  |  |  | Control group | Placebo | 2972 (43) | 4.9 (0.6) |
| Dutch TIA | The Netherlands | TIA or non-disabling ischaemic stroke <3 months before recruitment | Feb 1986 - Mar 1989 | All |  | 1473 (36) | 0.9 (0.9) |
|  |  |  |  | Treatment group | BETA (Atenolol) | 732 (34) | 0.8 (0.9) |
|  |  |  |  | Control group | Placebo | 741 (38) | 0.9 (0.9) |

**More versus less intensive treatment**

| Study | Country | Inclusion criteria | Randomisation period | Randomisation group | N participants (% women) | Mean follow-up duration, years (SD) |
| --- | --- | --- | --- | --- | --- | --- |
|  |  |  |  |  |  |  |
| ACCORD | Multi-country (USA/Canada) | Type 2 diabetes + CVD risk factors/40-74yrs + CVD | Jun 2003 - May 2006 | All | 4733 (48) | 4.7 (1.5) |
|  |  |  |  | Intensive (SBP <120) | 2362 (48) | 4.6 (1.5) |
|  |  |  |  | Standard (SBP <140) | 2371 (48) | 4.7 (1.5) |
| CARDIOSIS | Italy | ≥55yrs, CV risk factor | Feb 2005 - Feb 2007 | All | 1111 (59) | 2 (0.3) |
|  |  |  |  | Intensive (SBP <130) | 558 (59) | 2 (0.3) |
|  |  |  |  | Standard (SBP <140) | 553 (59) | 2 (0.2) |

AF, atrial fibrillation; CVD, cardiovascular disease; DBP, diastolic blood pressure; SBP, systolic blood pressure; TIA, transient ischaemic attack
