# Supplementary material for: Blood pressure-lowering treatment for the prevention of cardiovascular events in patients with atrial fibrillation: An individual participant data meta-analysis
Source: PLoS Med. 2021 Jun 1;18(6):e1003599. doi: 10.1371/journal.pmed.1003599 (PMC8168843; doi:10.1371/journal.pmed.1003599)
Supplement: S6 Table — (DOCX) [file pmed.1003599.s008.docx]

### S6 Table. Baseline characteristics of the participants included in atrial fibrillation meta-analyses stratified by trial

|  | **ACCORD** | **ACTIVE** | **ADVANCE** | **ALLHAT** | **ASCOT** | **CAPPP** | **CARDIOSIS** | **CASEJ** | **COLM** | **COPE** | **Dutch TIA** |
| --- | --- | --- | --- | --- | --- | --- | --- | --- | --- | --- | --- |
| n | 4733 | 9016 | 11140 | 33357 | 19257 | 10985 | 1111 | 4703 | 5141 | 3293 | 1473 |
| AGE (mean (SD)) | 62.73 (6.68) | 70.11 (9.73) | 65.77 (6.39) | 66.88 (7.71) | 63.00 (8.48) | 52.06 (8.38) | 66.99 (7.36) | 63.85 (10.54) | 73.62 (5.38) | 63.65 (10.72) | 64.43 (10.23) |
| SEX (Female (%)) | 2258 (47.7) | 3542 (39.3) | 4735 (42.5) | 15638 (46.9) | 4515 (23.4) | 5111 (46.5) | 653 (58.8) | 2106 (44.8) | 2488 (48.4) | 1624 (49.3) | 534 (36.3) |
| SBP (mean (SD)) | 139.00 (15.28) | 138.25 (17.40) | 145.02 (21.54) | 146.27 (15.64) | 164.01 (18.01) | 160.72 (20.02) | 158.27 (8.50) | 162.85 (14.18) | 157.99 (12.61) | 153.91 (11.56) | 157.28 (24.59) |
| DBP (mean (SD)) | 75.79 (9.97) | 82.39 (11.30) | 80.65 (10.93) | 84.02 (10.06) | 94.65 (10.37) | 98.95 (10.03) | 87.23 (8.08) | 91.70 (11.19) | 86.97 (10.79) | 88.80 (9.74) | 90.80 (11.96) |
| AF N (%) | 42 (0.9) | 9016 (100.0) | 847 (7.6) | 318 (1.0) | 230 (1.2) | 70 (0.6) | 0 (0.0) | 0 (0.0) | 0 (0.0) | 0 (0.0) | 0 (0.0) |
| IHD N (%) | 4083 (86.3) | 2294 (25.4) | 2380 (21.4) | 8415 (25.4) | 5284 (27.4) | 201 (1.8) | 128 (11.5) | 596 (12.7) | 563 (11.0) | 109 (3.3) | 138 (9.4) |
| CVD N (%) | 307 (6.5) | 1230 (13.6) | 1438 (12.9) | NA | 2121 (11.0) | 160 (1.5) | 91 (8.2) | 473 (10.1) | 751 (14.6) | 126 (3.8) | 1473 (100.0) |
| DM N (%) | 4733 (100.0) | 1785 (19.8) | 11140 (100.0) | 12063 (36.2) | 5145 (26.7) | 572 (5.2) | 0 (0.0) | 0 (0.0) | 1362 (26.5) | 466 (14.2) | 79 (5.4) |
| CKD N (%) | NA | NA | NA | NA | 12017 (62.4) | 28 (0.3) | 0 (0.0) | 2720 (57.8) | 108 (2.1) | 834 (32.1) | NA |
| SMOKING N (%) | 4107 (86.8) | 698 (7.7) | 1550 (13.9) | 7303 (36.7) | 6277 (32.6) | 2431 (22.1) | 226 (20.3) | 1025 (21.8) | 551 (10.8) | 700 (21.3) | 693 (47.0) |
| BMI (mean (SD)) | 32.15 (5.49) | 29.07 (5.77) | 28.34 (5.19) | 29.78 (11.27) | 28.72 (4.57) | 27.85 (4.39) | 27.82 (4.18) | 24.55 (3.66) | 24.27 (3.45) | 24.55 (3.39) | NA |

|  | **EWPHE** | **HIJCREATE** | **JMIC-B** | **NORDIL** | **ONTARGET** | **PROGRESS** | **SHEP** | **STOP2** | **SYSTEUR** | **TRANSCEND** | **VALUE** |
| --- | --- | --- | --- | --- | --- | --- | --- | --- | --- | --- | --- |
| n | 840 | 2049 | 1650 | 10881 | 25620 | 6105 | 4736 | 6614 | 4695 | 5926 | 15245 |
| AGE (mean (SD)) | 71.77 (8.03) | 65.30 (9.17) | 64.49 (8.51) | 59.90 (6.50) | 67.04 (7.20) | 63.90 (9.55) | 71.62 (6.70) | 76.03 (3.94) | 69.74 (6.70) | 67.48 (7.35) | 67.23 (8.13) |
| SEX (Female (%)) | 586 (69.8) | 405 (19.8) | 515 (31.2) | 5583 (51.4) | 6831 (26.7) | 1852 (30.3) | 2690 (56.8) | 4416 (66.8) | 3138 (66.8) | 2547 (43.0) | 6468 (42.4) |
| SBP (mean (SD)) | 182.63 (16.48) | 135.28 (18.00) | 146.16 (19.39) | 173.47 (17.62) | 141.82 (17.41) | 146.97 (19.01) | 170.30 (9.40) | 194.10 (15.29) | 173.85 (9.96) | 140.97 (16.63) | 154.65 (18.99) |
| DBP (mean (SD)) | 100.54 (7.10) | 75.70 (11.89) | 82.02 (11.74) | 105.74 (5.30) | 82.07 (10.40) | 85.68 (10.83) | 76.91 (8.30) | 97.84 (10.02) | 85.48 (5.87) | 81.89 (10.13) | 87.52 (10.79) |
| AF N (%) | 22 (2.6) | 135 (6.6) | 0 (0.0) | 101 (0.9) | 846 (3.3) | 476 (7.8) | 0 (0.0) | 313 (4.7) | 246 (5.2) | 205 (3.5) | 398 (2.6) |
| IHD N (%) | 73 (8.7) | 1745 (85.2) | 1650 (100.0) | 496 (4.6) | 19102 (74.6) | 983 (16.1) | 232 (4.9) | 647 (9.8) | 164 (3.5) | 4418 (74.6) | 6981 (45.8) |
| CVD N (%) | 63 (7.5) | 205 (10.0) | NA | 271 (2.5) | 5342 (20.9) | 5124 (83.9) | 66 (1.4) | 502 (7.6) | 124 (2.6) | 1302 (22.0) | 3014 (19.8) |
| DM N (%) | 72 (8.6) | 780 (38.1) | 372 (22.5) | 727 (6.7) | 9612 (37.5) | 761 (12.5) | NA | 719 (10.9) | 449 (9.6) | 2118 (35.8) | 4823 (31.6) |
| CKD N (%) | 2 (0.2) | 3 (0.1) | NA | 31 (0.3) | NA | NA | NA | NA | 20 (0.4) | NA | NA |
| SMOKING N (%) | 143 (17.0) | 509 (24.8) | 563 (34.1) | 2442 (22.4) | 3225 (12.6) | 1279 (21.0) | 602 (100.0) | 594 (9.0) | 343 (7.3) | 582 (9.9) | 3664 (24.0) |
| BMI (mean (SD)) | 26.39 (4.53) | 24.63 (2.99) | 24.04 (2.94) | 27.80 (4.34) | 28.16 (4.77) | 25.66 (3.78) | 27.11 (4.79) | 26.72 (4.00) | 27.03 (4.10) | 28.19 (4.82) | 28.63 (5.04) |

AF, atrial fibrillation; BMI, body mass index; BP, blood pressure; CKD, chronic kidney disease; CVD, cerebrovascular disease; DBP, baseline diastolic blood pressure; DM, diabetes mellitus; IHD, ischaemic heart disease; NA, not available; SBP, baseline systolic blood pressure

* Difference in average SBP reduction between trial arms at one year in mm Hg (positive values mean lower SBP in treatment arm and vice versa)
