# Supplementary material for: Blood pressure-lowering treatment for the prevention of cardiovascular events in patients with atrial fibrillation: An individual participant data meta-analysis
Source: PLoS Med. 2021 Jun 1;18(6):e1003599. doi: 10.1371/journal.pmed.1003599 (PMC8168843; doi:10.1371/journal.pmed.1003599)
Supplement: S7 Table — (DOCX) [file pmed.1003599.s009.docx]

### S7 Table. Sensitivity analyses including only trials that included patients with and without atrial fibrillation at baseline

| Major cardiovascular events | HR* | 95% CI | |
| --- | --- | --- | --- |
| One-stage model including only trials that included patients with and without atrial fibrillation at baseline (N=14) |  |  | |
| Atrial fibrillation | 0.94 | 0.83 | 1.06 |
| No atrial fibrillation | 0.91 | 0.89 | 0.94 |
| All (test for difference between subgroups p=0.68) | 0.91 | 0.89 | 0.94 |
|  |  |  |  |

* Standardised by 5-mmHg reduction in systolic blood pressure
