# Supplementary material for: Blood pressure-lowering treatment for the prevention of cardiovascular events in patients with atrial fibrillation: An individual participant data meta-analysis
Source: PLoS Med. 2021 Jun 1;18(6):e1003599. doi: 10.1371/journal.pmed.1003599 (PMC8168843; doi:10.1371/journal.pmed.1003599)
Supplement: S8 Table — (DOCX) [file pmed.1003599.s010.docx]

### S8 Table. Fixed and random effects two-stage meta-analyses

| Two-stage model using fixed effect meta-analysis | HR* | 95% CI |
| --- | --- | --- |
| Atrial fibrillation (I^2^ = 24%, p = 0.20) | 0.94 | [0.84 to 1.05] |
| No atrial fibrillation (I^2^ = 28%, p = 0.15) | 0.92 | [0.89 to 0.94] |
| All (I^2^ = 37%, p = 0.08) | 0.92 | [0.89 to 0.94] |
| Two-stage model using random effects meta-analysis |  |  |
| Atrial fibrillation (I^2^ = 19%, p = 0.20) | 0.91 | [0.79 to 1.06] |
| No atrial fibrillation (I^2^ = 40%, p = 0.15) | 0.91 | [0.87 to 0.95] |
| All (I^2^ = 45%, p = 0.08) | 0.91 | [0.87 to 0.95] |

* Standardised by 5-mmHg reduction in systolic blood pressure
