# Supplementary material for: Blood pressure-lowering treatment for the prevention of cardiovascular events in patients with atrial fibrillation: An individual participant data meta-analysis
Source: PLoS Med. 2021 Jun 1;18(6):e1003599. doi: 10.1371/journal.pmed.1003599 (PMC8168843; doi:10.1371/journal.pmed.1003599)
Supplement: S9 Table — (DOCX) [file pmed.1003599.s011.docx]

### S9 Table. Unadjusted effect of blood pressure-lowering treatment on primary and secondary outcomes, stratified by the presence of atrial fibrillation at baseline

|  | HR [95% CI] |
| --- | --- |
| Major cardiovascular events |  |
| AF | 0.94 (0.87 to 1.00) |
| No AF | 0.93 (0.90 to 0.96) |
| Overall | 0.93 (0.91 to 0.96) |
| p=0.693 |  |
| Stroke |  |
| AF | 0.88 (0.79 to 0.99) |
| No AF | 0.88 (0.84 to 0.92) |
| Overall | 0.88 (0.84 to 0.92) |
| p=0.90 |  |
| Ischaemic heart disease |  |
| AF | 0.97 (0.83 to 1.14) |
| No AF | 0.96 (0.92 to 0.99) |
| Overall | 0.96 (0.92 to 0.99) |
| p=0.872 |  |
| Heart failure |  |
| AF | 0.91 (0.82 to 1.01) |
| No AF | 0.93 (0.88 to 0.98) |
| Overall | 0.92 (0.88 to 0.97) |
| p=0.712 |  |
| Cardiovascular death |  |
| AF | 0.97 (0.89 to 1.07) |
| No AF | 0.94 (0.88 to 1.00) |
| Overall | 0.95 (0.90 to 1.00) |
| p=0.548 |  |
| All-cause death |  |
| AF | 1.02 (0.95 to 1.10) |
| No AF | 1.00 (0.97 to 1.03) |
| Overall | 1.00 (0.97 to 1.03) |
| p=0.50 |  |

AF: atrial fibrillation; HR: hazard ratio
