# Supplementary material for: Blood pressure-lowering treatment for the prevention of cardiovascular events in patients with atrial fibrillation: An individual participant data meta-analysis
Source: PLoS Med. 2021 Jun 1;18(6):e1003599. doi: 10.1371/journal.pmed.1003599 (PMC8168843; doi:10.1371/journal.pmed.1003599)
Supplement: S10 Table — (DOCX) [file pmed.1003599.s012.docx]

### S10 Table. Sensitivity analysis for the effect of blood pressure-lowering treatment on primary and secondary outcomes, stratified by the presence of atrial fibrillation at baseline, excluding the patients with the diagnosis of heart failure at baseline in ACTIVE-I trial

|  | Intervention | | Comparator | | HR* 95%CI |
| --- | --- | --- | --- | --- | --- |
|  | **Event** | **Total** | **Event** | **Total** |  |
| Major cardiovascular events |  |  |  |  |  |
| AF | 1278 | 5375 | 1237 | 5006 | 0.92 (0.84 to 1.02) |
| No AF | 10752 | 90869 | 10628 | 84435 | 0.92 (0.90 to 0.95) |
| Overall | 12030 | 96244 | 11865 | 89441 | 0.92 (0.90 to 0.95) |
| p=0.975 |  |  |  |  |  |
| Stroke |  |  |  |  |  |
| AF | 471 | 5375 | 474 | 5006 | 0.84 (0.72 to 0.98) |
| No AF | 3667 | 90869 | 3759 | 84435 | 0.87 (0.83 to 0.91) |
| Overall | 4138 | 96244 | 4233 | 89441 | 0.87 (0.83 to 0.91) |
| p=0.699 |  |  |  |  |  |
| Ischaemic heart disease |  |  |  |  |  |
| AF | 259 | 5375 | 241 | 5006 | 0.94 (0.78 to 1.14) |
| No AF | 5017 | 90869 | 4898 | 84435 | 0.94 (0.90 to 0.98) |
| Overall | 5276 | 96244 | 5139 | 89441 | 0.94 (0.91 to 0.98) |
| p=0.99 |  |  |  |  |  |
| Heart failure |  |  |  |  |  |
| AF | 436 | 5286 | 423 | 4923 | 0.94 (0.79 to 1.11) |
| No AF | 2397 | 76897 | 2389 | 70504 | 0.93 (0.88 to 0.99) |
| Overall | 2833 | 82183 | 2812 | 75427 | 0.94 (0.88 to 0.99) |
| p=0.976 |  |  |  |  |  |
| Cardiovascular death |  |  |  |  |  |
| AF | 531 | 5375 | 490 | 5006 | 0.83 (0.67 to 1.02) |
| No AF | 2134 | 90311 | 1896 | 83882 | 0.95 (0.89 to 1.01) |
| Overall | 2665 | 95686 | 2386 | 88888 | 0.94 (0.88 to 0.99) |
| p=0.221 |  |  |  |  |  |
| All-cause death |  |  |  |  |  |
| AF | 1039 | 5375 | 889 | 5006 | 1.04 (0.91 to 1.18) |
| No AF | 8641 | 90869 | 7904 | 84435 | 0.99 (0.96 to 1.03) |
| Overall | 9680 | 96244 | 8793 | 89441 | 1.00 (0.97 to 1.03) |
| p=0.533 |  |  |  |  |  |

AF: atrial fibrillation; HR: hazard ratio

* Standardised by 5-mmHg reduction in systolic blood pressure
