# Supplementary material for: Blood pressure-lowering treatment for the prevention of cardiovascular events in patients with atrial fibrillation: An individual participant data meta-analysis
Source: PLoS Med. 2021 Jun 1;18(6):e1003599. doi: 10.1371/journal.pmed.1003599 (PMC8168843; doi:10.1371/journal.pmed.1003599)
Supplement: S11 Table — (DOCX) [file pmed.1003599.s013.docx]

### S11 Table. Sensitivity analysis for the effect of blood pressure-lowering treatment on primary and secondary outcomes, stratified by the presence of atrial fibrillation at baseline, after adjustment for baseline systolic blood pressure, cardiovascular disease status and diabetes status at baseline

|  | Intervention | | Comparator | | HR* 95%CI |
| --- | --- | --- | --- | --- | --- |
|  | **Event** | **Total** | **Event** | **Total** |  |
| Major cardiovascular events |  |  |  |  |  |
| AF | 1837 | 6837 | 1837 | 6429 | 0.91 (0.83 to 1.00) |
| No AF | 10752 | 90869 | 10628 | 84435 | 0.92 (0.89 to 0.95) |
| Overall | 12589 | 97706 | 12465 | 90864 | 0.92 (0.89 to 0.95) |
| p=0.898 |  |  |  |  |  |
| Stroke |  |  |  |  |  |
| AF | 598 | 6837 | 622 | 6429 | 0.83 (0.72 to 0.97) |
| No AF | 3667 | 90869 | 3759 | 84435 | 0.85 (0.81 to 0.90) |
| Overall | 4265 | 97706 | 4381 | 90864 | 0.85 (0.81 to 0.90) |
| p=0.803 |  |  |  |  |  |
| Ischaemic heart disease |  |  |  |  |  |
| AF | 314 | 6837 | 296 | 6429 | 0.91 (0.76 to 1.09) |
| No AF | 5017 | 90869 | 4898 | 84435 | 0.93 (0.89 to 0.98) |
| Overall | 5331 | 97706 | 5194 | 90864 | 0.93 (0.89 to 0.97) |
| p=0.802 |  |  |  |  |  |
| Heart failure |  |  |  |  |  |
| AF | 698 | 6748 | 730 | 6346 | 0.89 (0.76 to 1.05) |
| No AF | 2397 | 76897 | 2389 | 70504 | 0.99 (0.92 to 1.07) |
| Overall | 3095 | 83645 | 3119 | 76850 | 0.96 (0.90 to 1.02) |
| p=0.257 |  |  |  |  |  |
| Cardiovascular death |  |  |  |  |  |
| AF | 865 | 6837 | 834 | 6429 | 0.90 (0.79 to 1.02) |
| No AF | 2134 | 90311 | 1896 | 83882 | 0.95 (0.89 to 1.01) |
| Overall | 2999 | 97148 | 2730 | 90311 | 0.95 (0.90 to 1.00) |
| p=0.482 |  |  |  |  |  |
| All-cause death |  |  |  |  |  |
| AF | 1471 | 6837 | 1335 | 6429 | 1.00 (0.91 to 1.11) |
| No AF | 8641 | 90869 | 7904 | 84435 | 0.98 (0.95 to 1.02) |
| Overall | 10112 | 97706 | 9239 | 90864 | 0.99 (0.96 to 1.02) |
| p=0.707 |  |  |  |  |  |

AF: atrial fibrillation; HR: hazard ratio

* Standardised by 5-mmHg reduction in systolic blood pressure
