# Supplementary material for: Blood pressure-lowering treatment for the prevention of cardiovascular events in patients with atrial fibrillation: An individual participant data meta-analysis
Source: PLoS Med. 2021 Jun 1;18(6):e1003599. doi: 10.1371/journal.pmed.1003599 (PMC8168843; doi:10.1371/journal.pmed.1003599)
Supplement: S12 Table — (DOCX) [file pmed.1003599.s014.docx]

### S12 Table. Sensitivity analysis for the effect of blood pressure-lowering treatment on primary and secondary outcomes, stratified by the presence of atrial fibrillation at baseline, excluding the trials with moderate risk of bias

|  | Intervention | | Comparator | | HR* 95%CI |
| --- | --- | --- | --- | --- | --- |
|  | **Event** | **Total** | **Event** | **Total** |  |
| Major cardiovascular events |  |  |  |  |  |
| AF | 1826 | 6789 | 1820 | 6385 | 0.93 (0.84 to 1.03) |
| No AF | 10332 | 85008 | 10160 | 78563 | 0.93 (0.90 to 0.95) |
| Overall | 12158 | 91797 | 11980 | 84948 | 0.93 (0.90 to 0.95) |
| p=0.897 |  |  |  |  |  |
| Stroke |  |  |  |  |  |
| AF | 595 | 6789 | 617 | 6385 | 0.83 (0.71 to 0.97) |
| No AF | 3492 | 85008 | 3534 | 78563 | 0.87 (0.83 to 0.92) |
| Overall | 4087 | 91797 | 4151 | 84948 | 0.87 (0.83 to 0.91) |
| p=0.555 |  |  |  |  |  |
| Ischaemic heart disease |  |  |  |  |  |
| AF | 313 | 6789 | 288 | 6385 | 0.98 (0.81 to 1.18) |
| No AF | 4817 | 85008 | 4710 | 78563 | 0.94 (0.90 to 0.98) |
| Overall | 5130 | 91797 | 4998 | 84948 | 0.94 (0.90 to 0.98) |
| p=0.665 |  |  |  |  |  |
| Heart failure |  |  |  |  |  |
| AF | 697 | 6734 | 729 | 6338 | 0.92 (0.77 to 1.09) |
| No AF | 2381 | 76495 | 2368 | 70088 | 0.95 (0.88 to 1.01) |
| Overall | 3078 | 83229 | 3097 | 76426 | 0.94 (0.88 to 1.00) |
| p=0.761 |  |  |  |  |  |
| Cardiovascular death |  |  |  |  |  |
| AF | 855 | 6789 | 825 | 6385 | 0.88 (0.76 to 1.02) |
| No AF | 1982 | 84450 | 1736 | 78010 | 0.94 (0.88 to 1.00) |
| Overall | 2837 | 91239 | 2561 | 84395 | 0.95 (0.90 to 1.00) |
| p=0.433 |  |  |  |  |  |
| All-cause death |  |  |  |  |  |
| AF | 1460 | 6789 | 1324 | 6385 | 1.02 (0.91 to 1.14) |
| No AF | 8327 | 85008 | 7582 | 78563 | 1.00 (0.96 to 1.03) |
| Overall | 9787 | 91797 | 8906 | 84948 | 1.00 (0.97 to 1.03) |
| p=0.675 |  |  |  |  |  |

AF: atrial fibrillation; HR: hazard ratio

* Standardised by 5-mmHg reduction in systolic blood pressure
