# Supplementary material for: Blood pressure-lowering treatment for the prevention of cardiovascular events in patients with atrial fibrillation: An individual participant data meta-analysis
Source: PLoS Med. 2021 Jun 1;18(6):e1003599. doi: 10.1371/journal.pmed.1003599 (PMC8168843; doi:10.1371/journal.pmed.1003599)
Supplement: S1 Methods — (DOCX) [file pmed.1003599.s015.docx]

# S1 Methods

# Supplementary Methods

### Systematic review

Trials were eligible for inclusion in the BPLTTC if they met one of the following criteria:

- Randomisation of patients between a BP lowering agent and a placebo or other inactive control (placebo-controlled trials);
- Randomisation of patients between different BP lowering intensities and/or targets (more versus less intense treatment trials); or
- Randomisation of patients between different antihypertensive drugs, including combinations (drug class comparison trials).

In addition, to avoid small study bias, trials were required to have a minimum of 1,000 patient-years of follow-up in each randomly allocated trial arm. Although no restrictions on publication date, setting or drugs were applied, trials that met the following criteria were excluded:

- Trials exclusively conducted in patients with HF or short-term interventions following acute myocardial infarction or other acute settings (e.g., acute stroke);
- Trials with non-pharmacological interventions of BP lowering without a drug comparison arm (e.g., trials of renal denervation);
- Trials without a clearly defined randomisation process.

### Identification of trials

A systematic review was conducted to identify trials that were eligible for inclusion in the current BPLTTC. A broad search query was run on electronic bibliographic databases, including PubMed/Medline (NCBI, Bethesda, MD, USA), The Cochrane Central Register of Controlled Trials (The Cochrane Collaboration, London, UK) and the ClinicalTrials.gov website covering the periods between the 1^st^ of January 1966 and the 1^st^ of June 2018. Filters were applied to restrict the search to RCTs or their meta-analyses. No language restrictions were applied. This search based on electronic databases was complemented with hand-searches of reference lists of eligible studies, related meta-analyses, and clinical trial registries to identify further relevant studies. The protocol for the systematic review, including details of the methods and search strategy, was registered with PROSPERO (CRD42018099283).

Records obtained from different sources were then screened, based on title and abstract. For studies that were potentially eligible, full manuscripts were retrieved and assessed for inclusion. Two independent reviewers performed study screening and selection in duplicate, with disagreements resolved by a third reviewer. **Figure 1** summarises the search strategy that underlies the BPLTTC.

### Data collection, transfer, and storage

For the final set of trials that complied with the aforementioned inclusion and exclusion criteria, investigators were invited to join the BPLTTC and share IPD. In addition, all existing BPLTTC collaborators were asked to provide additional IPD. As of May 2020, the collaboration had acquired data from 50 trials with about 350,000 participants. Despite ongoing efforts to gather IPD, there were 50 trials that could be potentially included in the BPLTTC for which IPD had not yet been obtained. Many of those trials were conducted many years ago and identification of the data guardian, or an electronic repository of the IPD, has proven challenging.

All data were transferred using a secure file transmission system, such as Oxford’s Oxfile system. Data has been stored in a secure server in the University of Oxford in keeping with data protection regulations and data sharing agreements. Access to the data is restricted to those persons directly involved in the research, and data can be used exclusively for the purpose of the proposed study.

### Data cleaning and harmonisation

We harmonised the IPD that were obtained for each trial before statistical analysis. Whenever there were discrepancies between the datasets that were shared with the collaboration and the information available in published articles, investigators were contacted for clarification. For missing data, investigators were also contacted to enquire about the possibility of providing those data. However, data were commonly unavailable, or investigators did not reply. In those circumstances, whenever possible, data were extracted from published reports.

### Details of the statistical modelling approach, subgroup analyses, and sensitivity analyses.

Since meta-analysis of IPD involves combining individual participant responses within each trial, the structure of the data is naturally hierarchical, with patients clustered within trials. Due to different settings and patient populations, the trials included in a meta-analysis are likely to have different baseline hazard functions and a less restrictive and more appropriate assumption of proportional hazards within each trial, rather than an overall baseline hazard function as in classical Cox regression models, can be achieved using stratified Cox regression models. We used fixed-effect IPD meta-analysis models, without any random or frailty effect term included in our models. Cox regression models with stratification by trial allowed us to account for the censoring of time-to-event data and for the clustering of subjects within trials by assuming different baseline hazards for each trial.^1^

According to the stratified Cox models, for the i^th^ individual in the j^th^ trial (i=1…n_j_, j=1…J), the hazard function at time t is estimated as follows:

$\lambda_{ij}\left( t \right)=\lambda_{0j}\left( t \right)exp(\beta_{1}\chi_{1ij}+\beta_{2}\chi_{2ij}+\beta_{3}\chi_{3ij})$ (1)

$\lambda_{ij}\left( t \right)=\lambda_{0j}\left( t \right)exp(\beta_{1}\chi_{1ij}+\beta_{2}\chi_{2ij}+\beta_{3}\chi_{3i}+\beta_{4}\chi_{4ij}+\beta_{5}\chi_{5ij})$ (2)

where

λ_0j_ is the baseline hazard function in the j_th_ trial

β_1_ indicates the log hazard ratio of the event in intervention group relative to the control group (which is assumed to be identical across trials)

β_2_ represent the coefficients for difference in SBP reduction between trial arms

β_3_ represent the coefficients for interaction between treatment and difference in SBP reduction between trial arms

β_4_ represent the coefficients for interaction between treatment and AF at baseline

β_5_ represent the coefficients for interaction between treatment and difference in SBP reduction between trial arms and AF at baseline

Models (1) and (2) were compared to estimate the interaction between treatment and AF at baseline using log-likelihood ratio tests.

All our models included (i) a term for treatment arm (intervention versus comparator as described in treatment comparisons), (ii) a term for the difference in SBP between arms aggregated at trial-level, used to standardise the estimates by 5-mm Hg SBP reduction, and (iii) an interaction term between treatment and difference in SBP. The inclusion of terms (ii) and (iii) aimed to take account of the differences in intensity of SBP reduction across trials. This is important because the different intensities of SBP reduction are assumed, in this case, to be the main drivers of heterogeneity in effects across trials. Although other methods have been used to standardise estimates for intensity of SBP reduction,^2, 3^ in one-stage IPD meta-analysis models the more appropriate method is to include this standardisation as part of the main model, by using meta-regression models adjusted for this variable. Moreover, this adjustment was performed using trial-level SBP reductions, calculated as differences between experimental and control arms for each trial, to maintain the randomised nature of comparisons and to lessen the impact of biases associated with post-randomisation variables adjustment (**Table S1**). The one-year time point was chosen because preliminary analysis showed that BP decreased linearly up to one year and then stabilised in both treatment and control arms. Although measurement of BP in AF is considered difficult, and the reliability of different methods and devices remains uncertain,^4, 5^ the difference in SBP reduction between treatment and control arms was identical within each trial for patients with and without AF at baseline. Therefore, the standardisation for the intensity of BP lowering was based on the difference in SBP reduction between treatment arms aggregated at trial level. As the average SBP reduction between arms amongst all trials was 3.7 mm Hg (due to inclusion of ‘head-to-head’ comparisons trials), we standardised the estimates for a 5-mmHg reduction in SBP.

Subgroup analyses were performed to investigate class-specific effects in trials that compared renin-angiotensin-aldosterone system (RAAS) inhibitor-based or calcium channel blocker (CCB)-based regimens versus placebo and/or standard treatment (beta-blocker and/or diuretic) (**Table S4**). The number of trials and AF participants available for other drug class comparisons was insufficient to perform further subgroup analyses (**Table S3**). For patients with AF at baseline, subgroup analysis was also performed according to baseline SBP considering it as a continuous variable and using a cut-off of 140 mmHg, as this is the treatment threshold recommended by contemporary hypertension guidelines.^6^ Wald tests were used to test for differences between subgroups. HR with 95% confidence intervals (CI) were reported and results were presented using forest plots with standardisation by 5 mmHg difference in SBP between trial arms.

Supplementary sensitivity analyses were performed (1) to compare one-stage with two-stage approaches; (2) to compare fixed effect and random effects models, (3) to investigate the effect of including trials that contributed to only one of the subgroups (i.e., either only included or excluded patients with baseline AF); (4) to investigate the effect of presenting treatment effects with adjustment for 3.7 mmHg, which was the average achieved BP difference between trial arms; (5) to assess the presence of acquisition bias using funnel plot and Egger’s regression test; (6) to check the sensitivity of findings after excluding the patients with the diagnosis of heart failure at baseline in ACTIVE-I trial; (7) to evaluate the effect of adjustment for SBP, diabetes and CVD status at baseline on the main findings and (8) to check the sensitivity of findings after excluding the trials with moderate risk of bias. For the two-stage meta-analyses, Cox regression models were fitted for each trial. Then, the estimates from each trial were combined using fixed-effect models with inverse variance weighing to calculate summary estimates with 95% CI. The analyses were standardised by 5 mm Hg reduction in SBP using a method that was applied in similar studies.^4,8^ The log of the summary statistic of each trial was multiplied by 5/delta (and the variance by (5/delta)^2^), where delta was the difference between the mean SBP reduction in the intervention and control arms for each trial. Heterogeneity between studies was quantified using I^2^ statistic and Cochran’s Q test.

# References

1. Smith CT, Williamson PR, Marson AG. Investigating heterogeneity in an individual patient data meta-analysis of time to event outcomes. Stat Med 2005;**24**(9):1307-19.

2. Cholesterol Treatment Trialists’ Collaboration. Efficacy and safety of statin therapy in older people: a meta-analysis of individual participant data from 28 randomised controlled trials. Lancet 2019;**393**(10170):407-415.

3. Ettehad D, Emdin CA, Kiran A, Anderson SG, Callender T, Emberson J, Chalmers J, Rodgers A, Rahimi K. Blood pressure lowering for prevention of cardiovascular disease and death: a systematic review and meta-analysis. Lancet 2016;**387**(10022):957-967.

4. Stergiou GS, Kollias A, Destounis A, Tzamouranis D. Automated blood pressure measurement in atrial fibrillation: a systematic review and meta-analysis. J Hypertens 2012;**30**(11):2074-82.

5. Halfon M, Wuerzner G, Marques-Vidal P, Taffe P, Vaucher J, Waeber B, Liaudet L, Ltaief Z, Popov M, Waeber G. Use of oscillometric devices in atrial fibrillation: a comparison of three devices and invasive blood pressure measurement. Blood Press 2018;**27**(1):48-55.

6. Williams B, Mancia G, Spiering W, Agabiti Rosei E, Azizi M, Burnier M, Clement DL, Coca A, de Simone G, Dominiczak A, Kahan T, Mahfoud F, Redon J, Ruilope L, Zanchetti A, Kerins M, Kjeldsen SE, Kreutz R, Laurent S, Lip GYH, McManus R, Narkiewicz K, Ruschitzka F, Schmieder RE, Shlyakhto E, Tsioufis C, Aboyans V, Desormais I, Group ESCSD. 2018 ESC/ESH Guidelines for the management of arterial hypertension. European Heart Journal 2018;**39**(33):3021-3104.
