# Supplementary material for: Blood pressure-lowering treatment for the prevention of cardiovascular events in patients with atrial fibrillation: An individual participant data meta-analysis
Source: PLoS Med. 2021 Jun 1;18(6):e1003599. doi: 10.1371/journal.pmed.1003599 (PMC8168843; doi:10.1371/journal.pmed.1003599)
Supplement: S1 Protocol — (DOCX) [file pmed.1003599.s017.docx]

**BPLTTC**

Study Protocol

Project: BASELINE_AF

Date and version: 7 July 2019, v 3.19

**Effects of blood pressure lowering treatment on cardiovascular outcomes in patients with atrial fibrillation: an individual-participant data meta-analysis**

**Working group members:**

Ana-Catarina Pinho-Gomes

Luis Azevedo

Emma Copland

Dexter Canoy

Eivind Berger

Johan Sundstrom

Mark Woodward

Kazem Rahimi

**Background**

Atrial fibrillation (AF) is the most common clinically significant cardiac arrhythmia and its incidence and prevalence are on the rise across the globe,[1] mainly due to population ageing and other cardiometabolic risk factors.[2] In observational studies, AF is associated with an increased risk of fatal and nonfatal cardiovascular events, such as stroke, ischaemic heart disease, heart failure and vascular dementia.[3] In trials that have mostly included patients without AF, blood pressure (BP) lowering treatment has been shown to be a highly effective strategy for reducing the risk of cardiovascular events.[4] However, whether this also applies to people with pre-existing AF remains uncertain. Randomised evidence from BP lowering trials in patients with AF is limited. The only randomised controlled trial (RCT) in patients with AF reported that an angiotensin receptor blocker did not reduce cardiovascular events, although this may be related to the relatively small BP reduction achieved.[5] As RCTs have typically included a small number of patients with established AF, it remains unclear whether BP lowering has an effect on cardiovascular outcomes or whether treatment effects would be different with other drug classes. To our knowledge, there are two trials at recruitment stage assessing the impact of an integrated care package for patients with AF, including among other interventions antihypertensive treatment (NCT03259893 and ChiCTR-OOC-17014138). In the meantime, pooled analysis of previous BP-lowering trials and stratification of their participants by presence or absence of AF at baseline offers an efficient and unbiased way of assessing of the effects of BP-lowering in patients with vs those without AF.

In this project, we, therefore, propose to use the large database of individual participant data (IPD) of BP lowering trials from the Blood Pressure Lowering Treatment Trialists’ Collaboration (BPLTTC) to address the question of the effect of BP lowering in patients with AF on cardiovascular outcomes.

**Aim**

- To compare the effect of antihypertensive treatment on fatal and non-fatal cardiovascular outcomes in patients with and without AF overall and by major drug classes and baseline BP.

**Methods**

**Study design**: Individual-participant data meta-analyses of BP lowering trials with assessment of treatment effects on cardiovascular disease and mortality endpoints by presence or absence of AF at baseline.

This study will use the resource provided by the Blood Pressure Lowering Treatment Trialists’ Collaboration (BPLTTC), which was initiated in 1995 as a collaboration between the Principal Investigators (PI) of major ongoing clinical trials of BP-lowering drugs.[6] Since its inception, the BPLTTC has provided reliable evidence on the benefits of BP reduction for prevention of major cardiovascular events and influenced international guidelines. This resource has been recently expanded to 50 trials with over 350,000 participants, offering an unprecedented opportunity to compare the effect of antihypertensive treatment in patients with and without AF at baseline. Information about AF status at baseline was sought for all trials that either published it or included that variable in the dataset provided to the collaboration.

**Eligibility criteria**

All RCTs of BP lowering treatment in BPLTTC will be eligible if they have collected information on presence of AF at baseline. Three types of trials will be eligible: (1) trials that included both patients with and without AF at baseline (n = 11); (2) trials that included only patients with AF at baseline (n = 1); and (3) trials that excluded patients with AF at baseline (n = 3). Information from additional trials is being sought.

**Definition of outcomes**

The primary outcome will be total major cardiovascular events, defined as the first occurrence of any of the following events:

1. Fatal or non-fatal stroke or cerebrovascular disease;
2. Fatal or non-fatal myocardial infarction or coronary artery disease;
3. Heart failure (HF) causing death or requiring hospitalization;

Secondary outcomes will include the individual elements of the composite endpoint as well as cardiovascular mortality and all-cause mortality. All outcomes will be based on the outcomes reported by primary trials.

**Treatment comparisons (Table 1)**

In addition to the analysis across all trials, supplementary analyses will be carried out to investigate class-specific effects in trials that compared different treatment regimens with placebo or standard treatment but not for trials of more versus less intensive BP reduction because there is currently only one trial in the latter category. In the broad group of trials comparing an active treatment with placebo, separate analyses will be performed for renin-angiotensin-aldosterone system (RAAS) inhibitor-based regimens versus placebo. In the broad group of trials comparing different active agents, separate analyses will be carried out for RAAS inhibitor-based regimens versus conventional treatment (diuretics and/or β-blocker (BB)-based regimens), and CCB-based regimens versus conventional treatment.

| Table 1: Treatment regimens in the included trials | | | | |
| --- | --- | --- | --- | --- |
| Trial | **Treatment 1** | **Treatment 2** | **Treatment 3** | **Control** |
| ACCORD | Intensive |  |  | Standard |
| EWPHE | Diuretic |  |  | Placebo |
| PROGRESS | ACEI-Diuretic |  |  | Placebo |
| ADVANCE | ACEI-Diuretic |  |  | Placebo |
| Syst-Eur | CCB |  |  | Placebo |
| ACTIVEI | ARB |  |  | Placebo |
| HIJ-CREATE | ARB |  |  | Placebo |
| CAPPP | ACEI |  |  | BB-Diuretic |
| NORDIL | CCB |  |  | BB-Diuretic |
| ASCOT | CCB-ACEI |  |  | BB-Diuretic |
| STOP H2 | ACEI | CCB |  | BB-Diuretic |
| COLM | ARB-CCB |  |  | ARB-Diuretic |
| COPE | CCB-ARB | CCB-BB |  | CCB-Diuretic |
| SHEP | BB-Diuretic | Reserpine-Diuretic |  | Placebo-Diuretic |
| LIFE | ARB |  |  | BB |
| ALLHAT | ACEI | CCB | Alpha-B | Diuretic |

**Data analysis**

Intention-to-treat analysis will be adopted using the data provided by each trial, after internal quality checks have been carried out to ensure that data are accurate and transferred without error.

For the analysis across all trials, intervention and control groups will be compared. For trials with two arms, the placebo or standard treatment arm will be considered as ‘control’ and the active treatment will be considered as ‘intervention’. For trials with three or more arms, patients in the treatment arms will be grouped together and compared with placebo or standard treatment arm.

For the treatment comparisons, different treatment arms within each trial will be considered separately and selected for specific comparisons. For trials with two arms, the placebo or standard treatment arm will be considered as ‘control’. For trials with three or more arms, patients in the placebo or standard treatment arm will be split in equal numbers between the intervention arms under investigation to avoid double counting of participants. In some trials, certain treatment arms may be excluded from this analysis as not all drug combinations will be analysed (as described above).

**Main analysis**

Treatment effects will be standardised for the difference in BP reduction between trial arms to account for the differences in intensity of BP reduction among trials. The BP difference will be calculated using a two-step approach. First, the mean BP reduction for each trial group will be calculated considering as achieved BP the value reported at one year or the closest to that time if missing. The one-year time point was chosen because preliminary analysis showed that BP decreases linearly up to one year and then stabilises. Second, the difference in the mean BP reduction between trial arms will be calculated. This variable will enter as covariate d in the one-stage models described below.

For the meta-analyses, a one-stage approach will be adopted, that is the IPD from all studies will be analysed simultaneously by adopting a single statistical model that accounts for clustering of participants within studies.[7-9] Therefore, the IPD from all studies will be analysed simultaneously by adopting a Cox regression model stratified by trial. In addition, to investigate the impact of baseline AF on treatment effect, baseline AF will be introduced as a covariate the model with an interaction with treatment arm.

Hazard ratios (HR) with 95% CI will be reported. Results will be presented using forest plots and standardised by 5 mmHg BP difference between treatment arms. Models with and without AF as covariate will be compared using the log-likelihood ratio test by means of analysis of variance (ANOVA), with χ^2^ test and respective p-values indicating whether there is a statistically significant interaction between baseline AF and treatment. If a significant interaction is found between baseline AF and treatment effect, treatment effects will be calculated for with and without AF at baseline.

Subgroup analysis will be performed for drug class as detailed in treatment comparisons and heterogeneity of treatment effect between subgroups will be assessed using χ^2^ tests.

Meta-regression will also be used to investigate the effect of the difference in the mean BP reduction between treatment arms on the relative risk reduction for the pre-defined outcomes. The results will be displayed using a scatter-plot with separate regression lines for subgroups of patients with and without AF.

All p-values will be calculated from two-tailed tests. Statistical analyses will be performed using the survival and metafor packages for R.

**Sensitivity analyses**

Sensitivity analyses will be performed: (1) to compare one-stage with two-stage approaches; (2) to investigate the impact of excluding trials that included only patients with or without baseline AF (i.e., including only trials that included both patients with and without AF at baseline).

For the two-stage meta-analyses, fixed effect models with inverse variance weighing using restricted maximum likelihood (REML) estimators will be used to calculate summary estimates with 95% CI for all outcomes. The analyses will be standardised by 5 mm Hg reduction in systolic BP using a method that has been applied in similar studies.[4, 10] The log of the summary statistic of each trial will be multiplied by 5/d (and the variance by (5/d)^2^), where d is the difference between the mean systolic blood BP reduction in the intervention and control arms in that trial. Heterogeneity between studies will be quantified using Cochran’s Q and I^2^ statistic with respective p-values. Meta-regression will be used if considerable residual heterogeneity remains after controlling for possible effect modifiers (e.g., baseline BP), with p-value adjusted for false positive findings based on Monte Carlo simulation.

**Sample size considerations**

With a total sample size of 100,000 participants, a known 14% prevalence of AF at baseline in the current trial sample, a 14% risk of incident CV events in participants without AF in the control arms, an approximate HR for CV events (fatal and non-fatal) of 0.80 for treatment versus control (based on previous BPLTTC analyses)[11], we estimate to have approximately 80% power to detect a statistically significant interaction of plus or minus 10% for an alpha of 0.05 (two-tailed). Assuming that the HR for total CV events (treatment versus control) in the population without baseline AF is 0.80, a 10% interaction translates into an HR for total CV events (treatment versus control) smaller than 0.70 or greater than 0.90 in participants with baseline AF. Therefore, if there is a clinically relevant interaction between baseline AF and the effect of BP-lowering treatment, we expect to have adequate power to detect it.

| **Table 2** | **Total N** | **AF N** |
| --- | --- | --- |
| **ACCORD** | 10251 | 127 |
| **ADVANCE** | 11140 | 845 |
| **CAPPP** | 10985 | 70 |
| **HIJ-CREATE** | 2050 | 135 |
| **NORDIL** | 10881 | 101 |
| **PROGRESS** | 6105 | 476 |
| **Syst-Eur** | 4695 | 246 |
| **ACTIVEI** | 9016 | 9016 |
| **SHEP** | 4736 | 0 |
| **COLM** | 5141 | 0 |
| **COPE** | 3293 | 0 |
| **STOP H2** | 6614 | 45 |
| **ASCOT** | 19257 | 230 |
| **EWPHE** | 840 | 22 |
| **Sum** | 105004 | 11313 |

**References**

1. Chugh SS, Havmoeller R, Narayanan K, Singh D, Rienstra M, Benjamin EJ, et al. Worldwide epidemiology of atrial fibrillation: a Global Burden of Disease 2010 Study. Circulation. 2014;129(8):837-47. Epub 2013/12/19. doi: 10.1161/circulationaha.113.005119. PubMed PMID: 24345399; PubMed Central PMCID: PMCPMC4151302.

2. Rahman F, Kwan GF, Benjamin EJ. Global epidemiology of atrial fibrillation. Nature reviews Cardiology. 2014;11(11):639-54. Epub 2014/08/13. doi: 10.1038/nrcardio.2014.118. PubMed PMID: 25113750.

3. Emdin CA, Anderson SG, Salimi-Khorshidi G, Woodward M, MacMahon S, Dwyer T, et al. Usual blood pressure, atrial fibrillation and vascular risk: evidence from 4.3 million adults. International journal of epidemiology. 2017;46(1):162-72. Epub 2016/05/05. doi: 10.1093/ije/dyw053. PubMed PMID: 27143136; PubMed Central PMCID: PMCPMC5407172.

4. Ettehad D, Emdin CA, Kiran A, Anderson SG, Callender T, Emberson J, et al. Blood pressure lowering for prevention of cardiovascular disease and death: a systematic review and meta-analysis. Lancet (London, England). 2016;387(10022):957-67. Epub 2016/01/03. doi: 10.1016/s0140-6736(15)01225-8. PubMed PMID: 26724178.

5. Yusuf S, Healey JS, Pogue J, Chrolavicius S, Flather M, Hart RG, et al. Irbesartan in patients with atrial fibrillation. The New England journal of medicine. 2011;364(10):928-38. Epub 2011/03/11. doi: 10.1056/NEJMoa1008816. PubMed PMID: 21388310.

6. Protocol for prospective collaborative overviews of major randomized trials of blood-pressure-lowering treatments. World Health Organization-International Society of Hypertension Blood Pressure Lowering Treatment Trialists' Collaboration. Journal of hypertension. 1998;16(2):127-37. Epub 1998/04/16. PubMed PMID: 9535138.

7. Burke DL, Ensor J, Riley RD. Meta-analysis using individual participant data: one-stage and two-stage approaches, and why they may differ. Statistics in medicine. 2017;36(5):855-75. Epub 2016/10/18. doi: 10.1002/sim.7141. PubMed PMID: 27747915; PubMed Central PMCID: PMCPMC5297998.

8. Debray TP, Moons KG, van Valkenhoef G, Efthimiou O, Hummel N, Groenwold RH, et al. Get real in individual participant data (IPD) meta-analysis: a review of the methodology. Research synthesis methods. 2015;6(4):293-309. Epub 2015/08/20. doi: 10.1002/jrsm.1160. PubMed PMID: 26287812; PubMed Central PMCID: PMCPMC5042043.

9. Riley RD, Lambert PC, Staessen JA, Wang J, Gueyffier F, Thijs L, et al. Meta-analysis of continuous outcomes combining individual patient data and aggregate data. Statistics in medicine. 2008;27(11):1870-93. Epub 2007/12/12. doi: 10.1002/sim.3165. PubMed PMID: 18069721.

10. Cholesterol Treatment Trialists’ Collaboration. Efficacy and safety of statin therapy in older people: a meta-analysis of individual participant data from 28 randomised controlled trials. Lancet (London, England). 2019;393(10170):407-15. Epub 2019/02/05. doi: 10.1016/s0140-6736(18)31942-1. PubMed PMID: 30712900; PubMed Central PMCID: PMCPMC6429627.

11. Czernichow S, Zanchetti A, Turnbull F, Barzi F, Ninomiya T, Kengne AP, et al. The effects of blood pressure reduction and of different blood pressure-lowering regimens on major cardiovascular events according to baseline blood pressure: meta-analysis of randomized trials. Journal of hypertension. 2011;29(1):4-16. Epub 2010/10/01. doi: 10.1097/HJH.0b013e32834000be. PubMed PMID: 20881867.
