# Supplementary material for: Blood pressure-lowering treatment for the prevention of cardiovascular events in patients with atrial fibrillation: An individual participant data meta-analysis
Source: PLoS Med. 2021 Jun 1;18(6):e1003599. doi: 10.1371/journal.pmed.1003599 (PMC8168843; doi:10.1371/journal.pmed.1003599)
Supplement: Attachment — Submitted filename: Answers_reviewers_PLOS_final.docx [file pmed.1003599.s018.docx]

**Response to requests from the editor**

*Although two reviewers are relatively positive about the paper, one reviewer (reviewer 2) raises substantial concerns about the selectivity of inclusion of data in this IPD meta, among other issues - for the paper to be considered potentially publishable in PLOS Medicine, the authors would need to come up with a convincing rebuttal to those points. In addition, although the authors' Discussion limitations section mentions some aspects of the analyses, the exact point raised by this reviewer does not seem to be mentioned there.

*Some minor style changes (to be addressed alongside the reviewers' points)

**Q1.** In the last sentence of the Abstract Methods and Findings section, please include a brief note about any key limitation(s) of the study's methodology.

**Reply**: We have now added a sentence about limitations. Please see below the revision:

*“Abstract*

*The findings of this study need to be interpreted in light of its limitations, such as the limited number of trials included, difficulty in ascertaining AF cases and measuring BP in patients with AF.”*

**Q2.** *At this stage, we ask that you include a short, non-technical Author Summary of your research to make findings accessible to a wide audience that includes both scientists and non-scientists. The Author Summary should immediately follow the Abstract in your revised manuscript. This text is subject to editorial change and should be distinct from the scientific abstract. Please see our author guidelines for more information: https://journals.plos.org/plosmedicine/s/revising-your-manuscript#loc-author-summary

**Reply**: The author summary has been included in the revised version of the manuscript.

**Q3.** We would ask that the authors state clearly in the Methods section of the paper whether the analysis presented here corresponds to one laid out in a prospectively-specified protocol or analysis plan? Please state this (either way) early in the Methods section.

**Reply**: This analysis followed a pre-specified plan that was, however, not published. We clarified this in the Methods. Please see below the added sentence:

*“This analysis followed a pre-specified protocol, that is provided as supplementary material.”*

**Q4.** If a prospective analysis plan (from your funding proposal, IRB or other ethics committee submission, study protocol, or other planning document written before analyzing the data) was used in designing the study, please include the relevant prospectively written document with your revised manuscript as a Supporting Information file to be published alongside your study, and cite it in the Methods section. A legend for this file should be included at the end of your manuscript.

**Reply**: We have now uploaded the protocol as a supplementary file and referred to it in the manuscript, as described above.

**Q5.** If no such document exists, please make sure that the Methods section transparently describes when analyses were planned, and when/why any data-driven changes to analyses took place.

**Reply**: We have now uploaded the protocol as a supplementary file and referred to it in the manuscript, as described above.

**Q6**. In either case, changes in the analysis-- including those made in response to peer review comments-- should be identified as such in the Methods section of the paper, with rationale.

**Reply**: We clarified in the Methods whenever analyses were altered as part of the peer-review process.

**Response to comments from the reviewers**

**Reviewer #1:**

This paper describes an individual participant data meta–analysis of blood pressure lowering and atrial fibrillation (AF).

I was asked for a statistical report and I interpret that to include all aspects of the design and conduct of the study.

Points of detail

**Q7**. Page 5 The terms standardised unfortunately has several meanings in statistical writing. I wonder whether it can be replaced here with, for instance, ‘Thus we present estimates for [. . . ] a 5–mm Hg reduction in SBP’.

**Reply**: We agree with the reviewer that term “standardised” could be confusing for the readers; therefore, we have changed the term “standardised” to “adjusted”, because it reflects more accurately what has been done in this analysis. We have thus removed the term “standardised” or “standardisation” throughout the manuscript, including figure legends.

**Q8.** Page 7, Table 1 There seems to be a huge difference between the AF and non–AF participants in smoking but the authors have made no comment about this. Have I misread the table?

**Reply**: This is correct; thank-you for pointing out this interesting difference. We have added a sentence to the Results commenting on this descriptive difference. We would point out that our main analysis first assesses treatment effects in each subgroup (AF vs no AF) and then compares relative risk reductions between these two groups. Thus, the imbalances in baseline characteristics are merely descriptive and to some extent expected given that higher risk profile of patients with AF compared with those without.

*“The prevalence of smoking was higher in patients with AF than in those without AF (9.3% versus 24.3%, respectively).”*

Points of more substance

Inclusion criteria

**Q9.** The authors have included trials with no participants or all participants with AF (page 6). This seems to me misguided.

Including the trial with all participants with AF is fine if the point of the review is to study the effect of treatment in people with AF without a direct comparison with the effect of treatment in people without AF. However, most of the article involves a comparison with people without AF and the analysis presented as a sensitivity analysis seems to me preferable for making comparisons between AF and non–AF. As far as I can see Figure 4 is the only AF–specific analysis and the others involve a comparison with non–AF.

I struggle to see the value of including people without AF in an article with an emphasis on AF.

I think I would only use the trials which allowed for a direct comparison as the main analysis and present the analysis including the AF-only data as the sensitivity analysis.

**Reply**: A priori, we chose one-stage meta-analysis as our primary method, for reasons expounded in the text (p6): *The one-stage approach has more power and flexibility than a two-stage approach to test for treatment-covariate interactions even when few studies are available (15, 16)*. We included RCTs that excluded patients with AF, as recommended for IPD meta-analysis using a one-stage approach and focused on treatment-covariate interactions[1], because those RCTs contribute to one of the strata of the covariate (Non-AF) assessed in this meta-analysis. Indeed, being able to do this is an important advantage of using a one-stage model for IPD meta-analysis. We used a stratified Cox regression model that takes account that patients come from different trials and assumes a different baseline hazard for each trial. This enables comparisons of patients included in different trials, even if the trial did not allow for estimating the interaction between AF and non-AF patients directly.

As you saw, for reassurance, we also performed a sensitivity analysis using a two-stage approach, which included only RCTs that allowed comparing patients with and without AF directly (i.e., RCTs that included both patients with and without AF). Reassuringly, the findings of this sensitivity analysis were comparable to the findings of the main analysis, although the confidence intervals were wider due to the smaller sample size.

Generalisability

**Q10.** What range of SBP is at issue here? I assume that in blood pressure lowering trials there must be some lower limit below which people are not eligible, although I am not an expert on cardiology. The article seems to issue a blanket recommendation that all people with AF should have their blood pressure lowered but is that based on evidence throughout the range? Suppose my GP wanted to provide me with an evidence–based recommendation as to whether I, with episodes of paroxysmal AF and systolic between 115 and 120, should take blood pressure lowering drugs could she use this as evidence?

**Reply**: Although most randomised clinical trials had an inclusion criterion of blood pressure in the hypertensive range at baseline, some trials (e.g., ADVANCE, PROGRESS) randomised patients irrespective of baseline blood pressure level. Therefore, our study included patients with baseline blood pressure below the recommended thresholds for diagnosing hypertension and starting treatment. Indeed, this is one of the key strengths of this study. Please see below the histogram showing the distribution of participants according to baseline systolic BP categories. As you can see in the plot, the study database included participants across a wide range of systolic blood pressure values at baseline. However, the clinical decision to treat lies between the patient and the clinician, given the full clinical picture available for that patient. Our data simply suggest that BP-lowering treatment is effective in reducing CVD risk even in patients with AF, with no evidence of a difference in relative effects at a cut-off of 140 mmHg baseline SBP.

**Figure. Distribution of systolic blood pressure in patients with atrial fibrillation at baseline.**

**Q11.** Summary

The authors have not completely convinced me.

**Reply**: We hope we have now reassured you that our analyses are appropriate and the conclusions supported by the results.

----------------------------------------------------------------------------------------------------------------------

**Reviewer #2:**

Dear Editors of PLOS Medicine,

Thank you for the opportunity to review this manuscript by Prof. Kazem Rahimi and colleagues. This is an individual-participant data meta-analysis from the Blood Pressure Treatment Trialists Collaboration (BPLTTC), aiming to assess if the effect of blood pressure (BP) lowering treatment differs between people with and without atrial fibrillation (AF), and if treatment effect in people with AF differs by baseline BP level and choice of antihypertensive agent.

Firstly, the authors should be complemented for the effort to update of the BPLTTC database. This will surely be of great importance to the field for years to come. Further, the manuscript is well written, with clear graphs and tables.

**Q12.** Unfortunately, I have several major concerns that needs to be addressed. Firstly, this meta-analysis does not build on a systematic review of the literature. This is problematic, because inclusion of certain (not all of the available) trials may provide biased results, not representative of the totality of the available evidence. For example, the five-year-old meta-analysis by Ettehad et al.1, with similar inclusion criteria, included 123 trials, whereas the updated BPLTTC database includes 50 trials, of which 22 are included in this analysis. Without an underlying systematic review, it is not possible to assess the generalizability of the reported findings. When undertaking such a massive effort as updating the BPLTTC dataset, it would be a small thing to perform an updated systematic review, trying to find all available trials, thereby being able to assess the representativeness of the trials providing IPD.

**Reply**: We would like to thank the reviewer for positive feedback on the paper.

On points of concern, this study was indeed based on a systematic review, and its protocol has already been published (reference #13: <https://bmjopen.bmj.com/content/9/5/e028698>). This systematic review served as the basis for reaching out to trialists from all eligible studies to join the BPLTTC. Processes for data acquisition in general and the analysis of the interaction between blood pressure-lowering treatment and AF at baseline in particular were described in the overall protocol paper (with further detailed provided in the study-specific protocol).

We agree that failure to obtain data from all trials may lead to acquisition bias (type of publication bias in IPD meta-analysis) which is an inherent limitation of IPD meta-analysis. On the other hand, assessment of treatment interaction among subgroups is more challenging when conventional tabular meta-analyses of published trial results are adopted. In addition, IPD meta-analysis tend to obtain additional unpublished data and provide the opportunity for data checking and updating, to assess reliability of findings. Despite our excessive efforts since 2014, not all trials could be included in BPLTTC. This is largely due to 3 reasons: for some older trials IPD has been lost or is not accessible; informed consent and data governance issues such as GDPR prohibit Sponsors to share data; or Investigators are unwilling to share because ongoing plans for their own analysis. Nonetheless, to our knowledge, the BPLTTC is now the largest ever established IPD resource of randomized trials and should be able to address important open questions of BP-lowering treatment, including heterogenous treatment effects as in the present study.

To provide reassurance to the reviewer and readers that this inherent limitation of large-scale IPD meta-analysis are highly unlikely to have biased our results, we provide three lines of evidence: (i) We compare the overall effect of BP-reduction on major CVD in our study with the most comprehensive tabular meta-analysis of published data that the reviewer refers to (Ettehad et al. Lancet 2015) and find consistent effects (HR for each 5 mmHg reduction in SBP in our study and Ettehad et al. 0.91 and 0.90, respectively). Thus, despite the lack of contribution of some trialists to the collaboration, the treatment effects seem almost identical when the two sets of (partially overlapping) trials are compared. (ii) We have conducted a two-stage “publication bias” test using funnel plot as a visual inspection of the bias and Egger’s regression test using trials included in this study. As the plot below shows, the funnel plot is clearly symmetrical, which speaks against substantial bias (T statistics = -1.43, df = 20, p-value = 0.1675, bias coefficient -0.68, standard error 0.48). (iii) Lastly, further reassurance for the absence of bias is provided by our sensitivity analysis which confirms that comparisons of patient outcomes (AF vs non-AF) within the same trials lead to similar results. These analyses collectively suggest that any data missingness is expected to have led to loss of statistical power, which we had consciously taken into account in our prespecified analysis plan (please see protocol).

**Figure. Funnel plot for assessment of publication (acquisition) bias on the effect of blood pressure reduction and risk of major cardiovascular event.**

**Q13.** Secondly, also relating to the fact that this is not a systematic review, the authors present no risk of bias assessment of the included trials. This is a major concern, primarily because it makes it impossible for the reader to appraise the validity of the results from the meta-analysis (a meta-analysis is only as good as the included trials, and this is not reported), but also because it is a deviation from the meta-analysis protocol, published last year.2

**Reply**: This is an excellent suggestion, thank you. We have assessed the risk of bias for all the trials included in this study and added it as supplementary data (please see Table S2 in the supplementary method). Due to focus of BPLTTC on high-quality large-scale nature trials, the risk of bias was low. We have also clarified this point in the Methods and Results sections.

*“Page 6, line 116.*

*Risk of bias assessment*

*Risk of bias was performed using the Rob 2 tool from the Cochrane Collaboration (Table S2).[14]*

*Page 7, line 157.”*

*All trials were considered to be at low risk of bias.”*

**Q14.** Thirdly, the main advantage of IPD in a meta-analysis setting is to better be able to select and group participants, based on individual characteristics. Used correctly, this will minimize ecological bias and confounding by differences in baseline covariates between groups. This paper hasn't used the available IPD as efficient as possible. For example, in the ACTIVE-I trial (I'll come back to that), contributing with 67% of AF participants in this meta-analysis, one third of participants had heart failure (HF) at baseline.3 These participants likely had lower BP and benefited more from treatment compared to non-HF participants; and would have been managed according to HF guidelines (not hypertension guidelines) in clinical practice. Including such participants in an IPD meta-analysis on the effects of blood pressure lowering treatment will I) bias the results towards more positive findings in people with AF and low BP, II) impair the clinical usefulness of the results

because they do not necessarily apply to non-HF patients.

**Reply**: HF status of ACTIVE-I participants has not been shared with the BPLTTC, which prohibits us from investigating the hypothesis of heterogeneity of treatment effects by HF-status. We have re-contacted the ACTIVE-I Investigators but have not been able to obtain the data thus far. However, we believe that the following lines of evidence speak against the findings being driven by HF patients who tend to have lower BP, and were more likely to be treated with non-randomised ACE-inhibitors in additional to the trial intervention of irbesartan (vs placebo): (i) The published ACTIVE-I report did not found any evidence of heterogeneity of treatment effects when participants were stratified by ACE-I use or the three categories of baseline BP (p for interaction 0.9 and 0.15, respectively for the primary outcome, and 0.58 and 0.36 for the secondary outcome, NEJM 2011; 364:928-938); (ii) to evaluate the effect of ACTIVE-I trial on the main estimation, we conducted a leave-one-out sensitivity analysis in which we excluded ACTIVE-I trial and recalculated the estimations. As the table below shows, except for wider confidence intervals, there is no material change after excluding this trial. This speaks against any particular subgroups (e.g., HF) in ACTIVE-I driving the results.

| **Table. Sensitivity analysis for the effect of blood pressure-lowering treatment on primary and secondary outcomes, stratified by the presence of atrial fibrillation at baseline, excluding the ACTIVE-I trial.** | | | | | |
| --- | --- | --- | --- | --- | --- |
| Baseline CVD | **Intervention** | | **Comparator** | | **HR 95%CI** |
|  | Event | Total | Event | Total |  |
| **Major cardiovascular events** |  |  |  |  |  |
| AF | 600 | 2319 | 543 | 1931 | 0.94 (0.83 to 1.06) |
| non AF | 10752 | 90869 | 10628 | 84435 | 0.92 (0.90 to 0.95) |
| **Overall** | 11352 | 93188 | 11171 | 86366 | 0.93 (0.90 to 0.95) |
|  |  |  |  |  |  |
| **Stroke** |  |  |  |  |  |
| AF | 219 | 2319 | 210 | 1931 | 0.81 (0.67 to 0.98) |
| non AF | 3667 | 90869 | 3759 | 84435 | 0.87 (0.83 to 0.91) |
| **Overall** | 3886 | 93188 | 3969 | 86366 | 0.87 (0.83 to 0.91) |
|  |  |  |  |  |  |
| **Ischaemic heart disease** |  |  |  |  |  |
| AF | 171 | 2319 | 160 | 1931 | 0.90 (0.72 to 1.12) |
| non AF | 5017 | 90869 | 4898 | 84435 | 0.94 (0.90 to 0.98) |
| **Overall** | 5188 | 93188 | 5058 | 86366 | 0.94 (0.90 to 0.98) |
|  |  |  |  |  |  |
| **Heart failure** |  |  |  |  |  |
| AF | 216 | 2230 | 179 | 1848 | 1.02 (0.83 to 1.24) |
| non AF | 2397 | 76897 | 2389 | 70504 | 0.93 (0.88 to 0.99) |
| **Overall** | 2613 | 79127 | 2568 | 72352 | 0.94 (0.88 to 1.00) |
|  |  |  |  |  |  |
| **Cardiovascular death** |  |  |  |  |  |
| AF | 196 | 2319 | 181 | 1931 | 0.83 (0.67 to 1.02) |
| non AF | 2134 | 90311 | 1896 | 83882 | 0.95 (0.89 to 1.01) |
| **Overall** | 2330 | 92630 | 2077 | 85813 | 0.94 (0.88 to 0.99) |
|  |  |  |  |  |  |
| **All-cause death** |  |  |  |  |  |
| AF | 520 | 2319 | 402 | 1931 | 1.04 (0.91 to 1.18) |
| non AF | 8641 | 90869 | 7904 | 84435 | 0.99 (0.96 to 1.03) |
| **Overall** | 9161 | 93188 | 8306 | 86366 | 1.00 (0.97 to 1.03) |
|  |  |  |  |  |  |

**Q15.** Fourthly, inclusion of trials in which all participants had AF, and trials where none of the included participants had AF, greatly increases the risk of ecological bias. If only trials including both AF and non-AF participants would have been included, AF and non-AF participants would have been judged according to the same eligibility criteria, received the same intervention and control, and the same follow-up procedures, including event adjudication etc. Differences in baseline characteristics would still have been present, but these would have been small, and at least trial-level factors would have been similar. In the current analysis, the majority of participants in the AF group comes from one trial (ACTIVE-I), which was limited to AF participants only. This results in massive differences in age, baseline BP level, and concomitant treatment between groups. Further, it means AF and non-AF participants in the meta-analysis have been judged according to different eligibility criteria (trial level), received different interventions, and different follow-up.

**Reply**: We accounted for this in the statistical analysis. We used a one-stage Cox regression model, stratified by trial, which assumed a different baseline hazard for each trial. Therefore, it accounted for differences in the populations included in each trial, irrespective of the subgroup they contributed to (i.e., AF versus non-AF). Furthermore, our regression models were adjusted for the intensity of systolic blood pressure reduction, aggregated at the trial level, to preserve the randomised nature of the comparisons. The adjustment for the intensity of blood pressure-lowering was based on the fact that blood pressure reduction has been compellingly shown to be the key mechanism of action of blood pressure-lowering drugs,[2] which then justified the choice for assuming a fixed treatment effect in the meta-analysis (most of the heterogeneity present is indeed due to differences in the intensity of blood pressure reduction between trials). In addition, as we explained in reply to **Q14**, excluding the ACTIVE-I did not change the findings.

Nevertheless, we of course agree that it would have been ideal to only have studies with AF and non-AF subjects to avoid the issue suggested. Hence, we included sensitivity analyses including only trials of that kind, using a two-stage approach (please see below table). Since this did not make a material difference to our estimates, whilst having wider confidence limits, we can conclude that our chosen one-stage approach, including “one stratum” studies, did not lead to bias, but improved precision.

| Table. One-stage model including only trials that included patients with and without atrial fibrillation at baseline (N=14) | | | |
| --- | --- | --- | --- |
| Major cardiovascular events | **HR** | **CI 95%** | |
| Atrial fibrillation | 0.94 | 0.83 | 1.06 |
| No atrial fibrillation | 0.91 | 0.89 | 0.94 |
| All (test for difference between subgroups p=0.68) | 0.91 | 0.89 | 0.94 |

**Q16.** Fifthly, several decisions and interpretations relating the statistical methods are questionable.

1.  The authors (correctly) state that "Due to different settings and patient populations, the trials included in a meta-analysis are likely to have different baseline hazard functions…" as a motivation for why they stratified their Cox model. In the next sentence they go on stating they chose fixed-effects model for their meta-analysis. This makes no sense, given that the fixed-effects model builds on the assumption of one common treatment effect in all trials. This seems careless rather than "parsimonious", which is the argument used in the supplementary methods section. Cornell et al. discuss how to estimate uncertainty in case of heterogeneity (which was present in the current analysis).4

**Reply**: We must distinguish differences in settings and populations, which are accounted for by differences in baseline hazards, from differences in treatment effects, which are mainly dependent on the randomised intervention. Stratified Cox models deal with the first difference (settings and populations), and random/fixed effects deal with the second (intervention effects). In this case, the intervention was BP lowering treatment and there is compelling evidence that the key mechanism of action of BP lowering drugs is BP reduction itself. The best evidence currently available, including a very recent network meta-analysis, demonstrates that the choice of drug for lowering BP is of token importance, provided that therapeutic regimen allows achieving the target BP reduction. Also, as previously described, our one-stage IPD meta-analysis model adjusts for differences in intensity of BP lowering; and that takes into account most of the heterogeneity observed between trials, as expected given the assumptions on which our analysis was based upon. Therefore, there is a strong rationale for assuming a fixed treatment effect in our meta-analysis of trials using different anti-hypertensive medications. Furthermore, the choice between fixed effect and random effects is largely a question of belief (given the well-known limitations of heterogeneity tests and analysis) on the existence of truly different effects that, in this particular case, we do not believe exist. We set out our a priori assumptions and goals in our protocol paper (<https://bmjopen.bmj.com/content/9/5/e028698>). Please be aware that this is not the same thing as saying that there are no differences between studies and studies’ settings. Indeed, those differences are obvious. Our argument and the basis of the statistical options for our IPD meta-analytical model is the fact that those differences are better accounted for by stratified Cox models and not necessarily by a random-effects model that we do not believe is more appropriate in this case (given our previously stated assumptions). For more details regarding this particular issue please see the reference 1 of our supplementary file, a paper by Smith et al. published in Statistics in Medicine in 2005 (Investigating heterogeneity in an individual patient data meta‐analysis of time to event outcomes. Statistics in Medicine, 2005, 24(9): 1307-1319; <https://pubmed.ncbi.nlm.nih.gov/15685717/>). In addition, for reassurance, in our sensitivity analysis using the two-stage meta-analysis method, we employed both fixed effect and random effects methods and there was no material difference in treatment effect estimates in patient with and without AF. We did not report those results in the original submission, but add them in our revision.

| Table. Fixed and random effect two-stage meta-analysis as sensitivity analysis | | | | |
| --- | --- | --- | --- | --- |
| Major cardiovascular events | | | | |
|  | **Fixed effect model** | | **Random effect model** | |
| Two-stage meta-analysis | **HR** | **CI 95%** | **HR** | **CI 95%** |
| Atrial fibrillation (I^2^ = 12%, p=0.31) | 0.92 | 0.84 to 1.01 | 0.92 | 0.83 to 1.02 |
| No atrial fibrillation (I^2^ = 34%, p=0.1) | 0.93 | 0.91 to 0.96 | 0.92 | 0.89 to 0.96 |

**Q17.** The authors "standardise" the results of each trial to 5 mmHg BP difference between groups. In the best-case scenario, this exaggerates the estimated treatment effect by about 30% (because the actual difference was 3.7 mmHg). In the worst-case scenario, this causes biased results due to shifts in weight between trials, as shown by Brunström et al. and discussed by Thomopoulos.5, 6

**Reply**:

We believe that the readers will more easily interpret a 5-unit measure than a 3.7-unit. Application of our results needs to be made in a general context, rather than in the context of the trials that we analysed. For reassurance, we performed sensitivity analysis adjusted by 3.7 mmHg and provided the forest plot as Supplementary data.

Our method is not flawed by dint of the issues raised in the referred papers which discussed issue on aggregate data meta-analysis, because we included the adjustment as part of the one-stage model using individual level data and also by different statistical framework. Nonetheless, to further support our reply, we have now calculated unadjusted (unstandardized) estimations. As you can see in the below table, there is no material change after using only unadjusted effect sizes (suggesting that the intensity of BP-lowering was roughly similar between subgroups).

-We have replaced the term “standardisation” by “adjustment” to avoid confusion and because the latter reflects better what was done.

| **Table. Unadjusted effect of blood pressure-lowering treatment on primary and secondary outcomes, stratified by the presence of atrial fibrillation at baseline.** | |
| --- | --- |
|  | **HR 95%CI** |
|  |  |
| **Major cardiovascular events** |  |
| AF | 0.94 (0.87 to 1.00) |
| non AF | 0.93 (0.90 to 0.96) |
| **Overall** | 0.93 (0.91 to 0.96) |
|  |  |
| **Stroke** |  |
| AF | 0.88 (0.79 to 0.99) |
| non AF | 0.88 (0.84 to 0.92) |
| **Overall** | 0.88 (0.84 to 0.92) |
|  |  |
| **Ischaemic heart disease** |  |
| AF | 0.97 (0.83 to 1.14) |
| non AF | 0.96 (0.92 to 0.99) |
| **Overall** | 0.96 (0.92 to 0.99) |
|  |  |
| **Heart failure** |  |
| AF | 0.91 (0.82 to 1.01) |
| non AF | 0.93 (0.88 to 0.98) |
| **Overall** | 0.92 (0.88 to 0.97) |
|  |  |
| **Cardiovascular death** |  |
| AF | 0.97 (0.89 to 1.07) |
| non AF | 0.94 (0.88 to 1.00) |
| **Overall** | 0.95 (0.90 to 1.00) |
|  |  |
| **All-cause death** |  |
| AF | 1.02 (0.95 to 1.10) |
| non AF | 1.00 (0.97 to 1.03) |
| **Overall** | 1.00 (0.97 to 1.03) |

**Q18.** At several occasions, the authors use underpowered analyses to motivate their choices of statistical methods, and to draw conclusions about possible interactions. For example, heterogeneity analyses should never be used to decide whether to use fixed- or random-effects model.7 Especially in situations like this, when the sample size of AF patients is relatively small, the test for heterogeneity will fail to detect fairly large differences in treatment effects because of the large standard error in the AF groups. Further, research question 3 and 4, regarding the possible interaction between baseline BP and treatment effect, and choice of agent and treatment effect, cannot be answered by the available data. As is evident from Figure 4 and 5, confidence intervals are wide, e.g. spanning from 0.76 to 1.06 for the <140 mmHg group in the BP analysis, reflecting the low number of participants and events. Indeed "no evidence for an association" is not the same as "evidence for no association"; this is not reflected in the authors' interpretations. Specifically, the conclusion that BP lowering is beneficial in AF patients, even below SBP 140 mmHg, stated in the abstract as well as the full text conclusion, is not supported by the data.

**Reply**: We agree that heterogeneity should not be used to determine whether fixed effect analyses should be used. As stated above, this was a pre-determined decision (see reference #13: …). Nevertheless, readers are likely to be interested in the degree of heterogeneity across studies, so it is reasonable to include such information.

As with the comment relating to research questions 3 and 4, we agree. We have changed the abstract as well as the Discussion and Conclusion to acknowledge that subgroup analyses based on baseline SBP and drug class were underpowered and, hence, no firm conclusions can be drawn. We also amended the discussion about BP thresholds and targets to clarify that subgroup analysis according to baseline BP had low power, and hence a clinically significant difference could not be ruled out.

*“Page 12, line 258.*

*Furthermore, our study suggested that patients with AF included in trials had a relatively low baseline SBP, with almost half of them not meeting the recommended threshold for treatment initiation of 140 mmHg.[3] Although we found no evidence that the relative risk reduction afforded by BP lowering differed above or below the 140 mmHg threshold, this analysis had low power and hence a clinically significant difference could not be ruled out.*

Minor issues:

**Q19.** Inclusion criteria are different in abstract and methods; 1000 patient-years per trial or treatment arm?

**Reply**: Thank-you. We have now corrected this. It should be 1000 patient-years follow-up in each randomised arm.

*“Page 2, line 33.*

*Individual participant data was extracted from all trials with over 1,000 patient-years of follow-up in each arm, which had randomly assigned patients to different classes of BP-lowering drugs, BP-lowering drugs vs placebo, or to more vs less intensive BP-lowering regimens.”*

**Q20.** Numbers in fig 4 does not add up. 2982+3566 ≠ 6837; 3091+3615 ≠ 6429 etc. Numbers in BP strata are not compatible with numbers in Fig 3.

**Reply**: We corrected the numbers in Figure 4.

1.            Ettehad D, Emdin CA, Kiran A, et al. Blood pressure lowering for prevention of cardiovascular disease and death: a systematic review and meta-analysis. Lancet 2016; 387: 957-67.

2.            Rahimi K, Canoy D, Nazarzadeh M, et al. Investigating the stratified efficacy and safety of pharmacological blood pressure-lowering: an overall protocol for individual patient-level data meta-analyses of over 300 000 randomised participants in the new phase of the Blood Pressure Lowering Treatment Trialists' Collaboration (BPLTTC). BMJ Open 2019; 9: e028698.

3.            Yusuf S, Healey JS, Pogue J, et al. Irbesartan in Patients with Atrial Fibrillation. New England Journal of Medicine 2011; 364: 928-38.

4.            Cornell JE, Mulrow CD, Localio R, et al. Random-Effects Meta-analysis of Inconsistent Effects: A Time for Change. Annals of Internal Medicine 2014; 160: 267-70.

5.            Brunström M, Carlberg B. Standardization according to blood pressure lowering in meta-analyses of antihypertensive trials: comparison of three methodological approaches. J Hypertens 2018; 36: 4-15.

6.            Thomopoulos C, Michalopoulou H. Outcome standardisation to blood pressure reduction in meta-analyses: sailing in uncharted waters. J Hypertens 2018; 36: 31-3.

7.            Cornell JE, Mulrow CD, Localio R, et al. Random-effects meta-analysis of inconsistent effects: a time for change. Ann Intern Med. 2014; 160(4):267-270.

-----------------------------------------------------------

Reviewer #3:

This study aimed to compare the effects of blood pressure (BP)-lowering drugs in patients with and without atrial fibrillation (AF) at baseline. The authors conclude that BP-lowering treatment reduces the risk of major cardiovascular events similarly in individuals with and without AF. Pharmacological BP lowering for prevention of cardiovascular events should be recommended in patients with AF even if BP is below the conventional 140-mmHg threshold.

This paper is generally nicely written and analysed

**Q21.**While there has not been a specific RCT in AF, some large observational studies on the optimal BP targets have been published eg from the Yonsei group in S Korea (Joung B and team).

These should be discussed in the Discussion, notwithstanding that observational cohorts are not a substitute for RCTs.

**Reply**: We added a comment on observational evidence and cited that large cohort study in the Discussion.

**Q22.**Figure 4 is simplistic - BP is not a yes/no parameter - show the impact on outcomes with BP as a continuous variable, for stroke, major CV events, all cause mortality etc

**Reply**: We agree, our subgroup analysis by baseline SBP was underpowered and so we could not perform such detailed analysis in patients with baseline AF. Indeed, we do not have enough number of cases and outcomes to conduct more detailed subgroup analysis with more detailed SBP categories. The best approach to have a notion about the effect across baseline SBP was dividing the SBP into two categories of below and above the typical threshold for hypertension diagnosis.

**Q23.** Figure 5 should how lump together 'placebo or BB/Diu'

Ideally show a summary forest plot of the different drug classes vs placebo, even to illustrate the effect sizes. Forest plot can also show inter-drug class comparisons

**Reply**: We agree that it would be interesting to compare different drug classes against placebo. However, as shown in Table S3 in Supplementary data, there was only one trial for comparisons between drugs and placebo, or equivalent drugs. Therefore, a more detailed subgroup analysis according to drug class was not possible to carry out. We have acknowledged that in the Supplementary Methods and also in the limitations.

*“Supplementary Data, page 3:*

*The number of trials and AF participants available for other drug class comparisons was insufficient to perform further subgroup analyses (****Table S3****).*

*Page 13, line 301.*

*Third, although it would have been interesting to compare the effects of other drug classes, particularly in head to head comparisons, only a small fraction of the trials included in the BPLTTC reported AF at baseline and were thus eligible for this study.”*

**References**

1.	Debray TP, Moons KG, van Valkenhoef G, Efthimiou O, Hummel N, Groenwold RH, et al. Get real in individual participant data (IPD) meta-analysis: a review of the methodology. Research synthesis methods. 2015;6(4):293-309. Epub 2015/08/20. doi: 10.1002/jrsm.1160. PubMed PMID: 26287812; PubMed Central PMCID: PMCPMC5042043.

2.	Wei J, Galaviz KI, Kowalski AJ, Magee MJ, Haw JS, Narayan KMV, et al. Comparison of Cardiovascular Events Among Users of Different Classes of Antihypertension Medications: A Systematic Review and Network Meta-analysis. JAMA Netw Open. 2020;3(2):e1921618. Epub 2020/02/23. doi: 10.1001/jamanetworkopen.2019.21618. PubMed PMID: 32083689; PubMed Central PMCID: PMCPMC7043193.

3.	Williams B, Mancia G, Spiering W, Agabiti Rosei E, Azizi M, Burnier M, et al. 2018 ESC/ESH Guidelines for the management of arterial hypertension. European Heart Journal. 2018;39(33):3021-104. doi: 10.1093/eurheartj/ehy339.
