# Supplementary material for: Blood pressure-lowering treatment for the prevention of cardiovascular events in patients with atrial fibrillation: An individual participant data meta-analysis
Source: PLoS Med. 2021 Jun 1;18(6):e1003599. doi: 10.1371/journal.pmed.1003599 (PMC8168843; doi:10.1371/journal.pmed.1003599)
Supplement: Attachment — Submitted filename: AF reviewer comment_Final_KR.docx [file pmed.1003599.s019.docx]

-----------------------------------------------------------

Requests from the editors:

In revising the manuscript for further consideration, your revisions should address the specific points made by each reviewer and the editors. Regarding comments from reviewer 2, we ask that you include additional covariates in your model/sensitivity analyses to alleviate the concerns noted by the reviewer or clearly outline the assumptions made and their limitations.

**Reply:** We appreciate the effort the reviewer has gone to in helping to improve this paper. We have now performed an additional sensitivity analysis as per reviewer’s request and have added it to our supplement. While this only provides further support for the validity of our findings, we believe that the rationale for such adjusted analyses put forward by the reviewer are not applicable to the hypothesis that we are testing. This is explained in our response to the reviewer’s comment.

Comments from the reviewers:

Reviewer #1: The authors have addressed my points. I still think it is a shame that we do not know whether there is a threshold below which further BP reduction is pointless but their data shows that this is only a minority interest.

**Reply**: Thank you for your positive feedback. The question of heterogeneity of treatment effects across the spectrum of blood pressure is of high clinical and scientific importance. Indeed, this is subject to a separate BPLTTC project. The preliminary results of that project were recently presented as a hotline late-breaker at the European Society of Cardiology. In brief, there was no evidence that baseline systolic BP modified treatment effects down to the normal systolic BP range (<120 mmHg).

Michael Dewey

Reviewer #2: Dear Editors of PLOS Medicine,

It is with great interest that I read the revised version of "Blood pressure lowering treatment for prevention of cardiovascular events in patients with atrial fibrillation: an individual-participant data meta-analysis" by Prof. Rahimi et al.

Firstly, I must emphasize that I think this meta-analysis is of huge importance clinically. Before, guidelines have only had the ACITVE-I trial and observational data to lean on, giving advice on BP lowering in atrial fibrillation. Thus, the current BPLTTC analysis will have huge impact, and therefore it needs to be great. For simplicity, I will follow the same structure as in my previous set of comments.

**Reply:** We appreciate this.

1) Ok, so it builds on a systematic literature search (not the same as systematic review). This should be reported in the manuscript (or appendix), not only referred to the published protocol. The authors should consider a PRISMA flowchart like the one in the protocol, but with an additional level below the 100 trials eligible for consideration, explaining what they explained in text in their response. Also, the funnel plot they use as an argument against acquisition bias should be added to the appendix. A systematic review needs to be 100% transparent.

**Reply**: We have now added the PRISMA flowchart and funnel plot to the paper (supplementary Figures S1 and S2) and these are shown below.

**Figure S1:** PRISMA diagram for included trials

**Figure S2.** Funnel plot for assessment of publication (acquisition) bias on the effect of blood pressure reduction and risk of major cardiovascular event.

We have conducted a two-stage publication bias test using funnel plot as a visual inspection of the bias and Egger’s regression test using trials included in this study. As the plot below shows, the funnel plot is clearly symmetrical, which speaks against substantial bias (T statistics = -1.43, df = 20, p-value = 0.1675, bias coefficient -0.68, standard error 0.48).

2) I´m very happy that the authors decided to perform risk of bias assessments. However, these are not included in the appendix submitted. It does sound strange, though, that all trials were judged to be at low risk of bias. For example, randomization in the CAPPP trial is known to have failed, as pointed out elegantly by Richard Peto in a letter to the editor few months after the original publication (<https://doi.org/10.1016/S0140-6736(05)75340-X>). In EWPHE, 36% of participants stopped the double-blind phase early; 15% of participants were lost to follow-up for non-mortality outcomes. The number of participants lost to follow-up widely exceeded the number of participants with cerebrovascular events (all in the original Lancet publication). How do the authors motivate "low risk of bias" in these circumstances? I really hope I´ll get to see the full risk of bias table, including judgements for each domain, in a future submission.

**Reply**: Please see the details of the revised risk assessment in supplementary Table S2 (and shown below).

**Table S2.** Assessment of risk of bias

| Trial | Randomization process | Deviations from intended interventions | Missing outcome data | Measurement of the outcome | Selection of the reported result | Overall |
| --- | --- | --- | --- | --- | --- | --- |
| ACCORD | Low | Low | Low | Low | Low | Low |
| ACTIVE-I | Low | Low | Low | Low | Low | Low |
| ADVANCE | Low | Low | Low | Low | Low | Low |
| ALLHAT | Low | Low | Low | Low | Low | Low |
| ASCOT | Low | Low | Low | Low | Low | Low |
| CAPPP | Moderate | Low | Low | Low | Low | Moderate |
| CARDIO-SIS | Low | Low | Low | Low | Low | Low |
| CASE-J | Low | Low | Low | Low | Low | Low |
| COLM | Low | Low | Low | Low | Low | Low |
| COPE | Low | Low | Low | Low | Low | Low |
| DUTCH-TIA | Low | Low | Low | Low | Low | Low |
| EWPHE | Low | Moderate | Low | Low | Low | Moderate |
| HIJCREATE | Low | Low | Low | Low | Low | Low |
| JMICB | Low | Low | Low | Low | Low | Low |
| NORDIL | Low | Low | Low | Low | Low | Low |
| ONTARGET | Low | Low | Low | Low | Low | Low |
| PROGRESS | Low | Low | Low | Low | Low | Low |
| SHEP | Low | Low | Low | Low | Low | Low |
| STOP-2 | Low | Low | Low | Low | Low | Low |
| SYSTEUR | Low | Low | Low | Low | Low | Low |
| TRANSCEND | Low | Low | Low | Low | Low | Low |
| VALUE | Low | Low | Low | Low | Low | Low |

Additionally, we conducted a sensitivity analysis excluding the trials with moderate risk of bias (CAPPP, EWPHE). We did not find any material change after excluding these trials (given their relatively low weight to the analyses). The results of this sensitivity analysis are shown below and can be found in the supplementary Table S12. A comment has been added to the results: “No material change was also seen after excluding the trials with moderate risk of bias.”

### **Table S12**. Sensitivity analysis for the effect of blood pressure-lowering treatment on primary and secondary outcomes, stratified by the presence of atrial fibrillation at baseline, excluding the trials with moderate risk of bias.

|  | Intervention | | Comparator | | HR* 95%CI |
| --- | --- | --- | --- | --- | --- |
|  | **Event** | **Total** | **Event** | **Total** |  |
| Major cardiovascular events |  |  |  |  |  |
| AF | 1826 | 6789 | 1820 | 6385 | 0.93 (0.84 to 1.03) |
| No AF | 10332 | 85008 | 10160 | 78563 | 0.93 (0.90 to 0.95) |
| Overall | 12158 | 91797 | 11980 | 84948 | 0.93 (0.90 to 0.95) |
| Stroke |  |  |  |  |  |
| AF | 595 | 6789 | 617 | 6385 | 0.83 (0.71 to 0.97) |
| No AF | 3492 | 85008 | 3534 | 78563 | 0.87 (0.83 to 0.92) |
| Overall | 4087 | 91797 | 4151 | 84948 | 0.87 (0.83 to 0.91) |
| Ischaemic heart disease |  |  |  |  |  |
| AF | 313 | 6789 | 288 | 6385 | 0.98 (0.81 to 1.18) |
| No AF | 4817 | 85008 | 4710 | 78563 | 0.94 (0.90 to 0.98) |
| Overall | 5130 | 91797 | 4998 | 84948 | 0.94 (0.90 to 0.98) |
| Heart failure |  |  |  |  |  |
| AF | 697 | 6734 | 729 | 6338 | 0.92 (0.77 to 1.09) |
| No AF | 2381 | 76495 | 2368 | 70088 | 0.95 (0.88 to 1.01) |
| Overall | 3078 | 83229 | 3097 | 76426 | 0.94 (0.88 to 1.00) |
| Cardiovascular death |  |  |  |  |  |
| AF | 855 | 6789 | 825 | 6385 | 0.88 (0.76 to 1.02) |
| No AF | 1982 | 84450 | 1736 | 78010 | 0.94 (0.88 to 1.00) |
| Overall | 2837 | 91239 | 2561 | 84395 | 0.95 (0.90 to 1.00) |
| All-cause death |  |  |  |  |  |
| AF | 1460 | 6789 | 1324 | 6385 | 1.02 (0.91 to 1.14) |
| No AF | 8327 | 85008 | 7582 | 78563 | 1.00 (0.96 to 1.03) |
| Overall | 9787 | 91797 | 8906 | 84948 | 1.00 (0.97 to 1.03) |

* Standardised by 5-mmHg reduction in systolic blood pressure

3) It is very unfortunate that HF data has not been shared since the HF is such an important confounder in BP lowering trials. The authors have done what they can with this comment, although the sensitivity analysis presented in this response must also be added to the appendix.

**Reply**: Fortunately, we have been able to obtain the information about participant’s HF status at baseline in ACTIVE-I trial now. This has enabled us to perform a sensitivity analysis excluding participants with a known diagnosis of HF. As previously suspected and indirectly inferred, this analysis rejects the hypothesis that the observed treatment effects are confined to those with HF only. The results of this sensitivity analysis are shown in the supplementary Table S10 and reproduced below.

**Table S10**. Sensitivity analysis for the effect of blood pressure-lowering treatment on primary and secondary outcomes, stratified by the presence of atrial fibrillation at baseline, excluding the patients with the diagnosis of heart failure at baseline in ACTIVE-I trial.

|  | Intervention | | Comparator | | HR* 95%CI |
| --- | --- | --- | --- | --- | --- |
|  | **Event** | **Total** | **Event** | **Total** |  |
| Major cardiovascular events |  |  |  |  |  |
| AF | 1278 | 5375 | 1237 | 5006 | 0.92 (0.84 to 1.02) |
| No AF | 10752 | 90869 | 10628 | 84435 | 0.92 (0.90 to 0.95) |
| Overall | 12030 | 96244 | 11865 | 89441 | 0.92 (0.90 to 0.95) |
| Stroke |  |  |  |  |  |
| AF | 471 | 5375 | 474 | 5006 | 0.84 (0.72 to 0.98) |
| No AF | 3667 | 90869 | 3759 | 84435 | 0.87 (0.83 to 0.91) |
| Overall | 4138 | 96244 | 4233 | 89441 | 0.87 (0.83 to 0.91) |
| Ischaemic heart disease |  |  |  |  |  |
| AF | 259 | 5375 | 241 | 5006 | 0.94 (0.78 to 1.14) |
| No AF | 5017 | 90869 | 4898 | 84435 | 0.94 (0.90 to 0.98) |
| Overall | 5276 | 96244 | 5139 | 89441 | 0.94 (0.91 to 0.98) |
| Heart failure |  |  |  |  |  |
| AF | 436 | 5286 | 423 | 4923 | 0.94 (0.79 to 1.11) |
| No AF | 2397 | 76897 | 2389 | 70504 | 0.93 (0.88 to 0.99) |
| Overall | 2833 | 82183 | 2812 | 75427 | 0.94 (0.88 to 0.99) |
| Cardiovascular death |  |  |  |  |  |
| AF | 531 | 5375 | 490 | 5006 | 0.83 (0.67 to 1.02) |
| No AF | 2134 | 90311 | 1896 | 83882 | 0.95 (0.89 to 1.01) |
| Overall | 2665 | 95686 | 2386 | 88888 | 0.94 (0.88 to 0.99) |
| All-cause death |  |  |  |  |  |
| AF | 1039 | 5375 | 889 | 5006 | 1.04 (0.91 to 1.18) |
| No AF | 8641 | 90869 | 7904 | 84435 | 0.99 (0.96 to 1.03) |
| Overall | 9680 | 96244 | 8793 | 89441 | 1.00 (0.97 to 1.03) |

* Standardised by 5-mmHg reduction in systolic blood pressure

4)            This is still my main concern, which I seem to be sharing with reviewer 1 (statistical reviewer). Of course, it is possible to include trials without AF participants in the statistical model, but including them will inevitably make AF and non-AF participants in the meta-analysis more different, as I explained in my previous comment. The authors state that this is accounted for, because the model assumes different baseline hazards for each trial, and because treatment arm, trial-level BP differences between treatment arms, and treatment-BP difference interaction, were included as covariates. For such a model to be valid, baseline hazard and BP difference would have to be the only two determinants of treatment effect beyond AF status, under study here. This implicitly assumes that no other trial or patient characteristics are important per se; everything is summarized in the baseline hazard function. This is a quite strong assumption, which, although the authors have argued for it previously, is not agreed upon by all in the hypertension community. For example, Thomopoulos et al (DOI:10.1097/HJH.0000000000001276 ) have found differences in treatment effect between people with and without diabetes, and Brunström et al have found differences between different baseline BP levels in primary prevention, and differences between primary and secondary preventive participants at low baseline BPs (doi:10.1001/jamainternmed.2017.6015). These meta-analyses are of course trial-level analyses, with potential for ecological bias, but nonetheless they are potential arguments for why other things (like DM, baseline BP, and previous CVD), beyond baseline hazard and BP difference, would be important to consider in a model accounting for differences between AF and non-AF participants.

Based on the difficulties described above, which would be much less severe had non-AF trials been excluded, I think the main analysis should be restricted to trials including both AF and non-AF participants. I completely disagree with the authors´ statement that their one-stage model "did not lead to bias, but improved precision". In my view, that improved precision is false because it comes from trials not including people with the condition under study. This, in turn, is very important because the evidence for AF participants is in fact weaker than suggested by this study.

If the authors persist including non-AF trials, and the editors chose to accept this (against my recommendation), I would recommend including more covariates (at least the ones mentioned above) in their model to fully account for potential differences in treatment effect between AF and non-AF participants. If the authors persist with the same model, and the editors chose to accept this (against my recommendation), they need to be very explicit about the assumptions that comes with that model, and discuss this under the limitations section.

**Reply**: We appreciate the remakable efforts made by the reviewer to help improve the quality of our paper and to check its validity. We have now conducted the additional sensitivity analysis that the reviewer feels necessary. As the table below indicates, these additional adjustments for systolic BP, CVD status and diabetes status at baseline had almost no effect on the estimated hazard ratios, thus supporting our conclusion that treatment effects were similar in people with or without AF at baseline.

However, we wish to respectfully contest the reviewer’s opinion on the appropriateness of our study design and the prespecified approach. The reviewer feels that our main approach is invalid because we falsely assume that there are no important trial- or individual-level characteristics other than baseline hazard and trial-level BP differences for estimation of treatment effects and their heterogeneity. We do not make such an assumption (and have previously explained our rationale for including baseline hazard and BP differences in the model). Indeed, as our results in Table 1 indicate, we report several differing clinical characteristics between people with or without AF. However, where we disagree is whether such differences should be adjusted for in the models. Although, from a methodological point of view, the favoured approach by the reviewer, namely to adjust for several additional baseline characteristics, might make sense, in our view it would be an overadjustment that would lead to testing a different hypothesis than the one posed by us. This would also render our results less clinically relevant. More specifically, our aim was to answer the question of whether a typical patient with AF as included in the trials would benefit similarly from BP lowering treatment as someone without AF. By contrast, the proposed approach by the reviewer is to ensure that patients with AF and no-AF are as similar as possible, which presumably will help understand the independent effect of AF on effect modification (as is commonly the case in mechanistic studies of individual risk factors). However, from clinical point of view, it is questionable whether there is any added value from isolating the effect of a single phenotype from all that typically goes with it. The phenotype ‘AF’ does not exist in isolation and it has many differing (and overlapping) features with the phenotype ‘no-AF’. For instance, people with AF are more likely to be older, to have HF or to be taking antiarrhythmic drugs. Adjusting for such differences might answer the question as to whether AF per se modifies treatment effects but not whether the average clinical trial patient with AF will have similar benefits from treatment to those without AF. The approach that we take is common practice when reporting subgroup analyses in clinical trials, of course with the well-known limitation of the ‘reference-class’ problem (e.g., see BMJ 2018;364:k4245 doi: 10.1136/bmj.k4245). By taking this approach and showing that no effect modification exists among patients grouped by presence or absence of a single characteristic, we inform clinical decision making and conclude that AF status cannot justify withholding (or initiation) of BP lowering treatment. We acknowledge that this approach suffers from the usual limitation of external validity in clinical trials. However, the approach proposed by the reviewer would only aggravate this and render the findings even less relevant to clinical practice (but perhaps generate some mechanistic insights). We had considered these issues carefully when finalising the protocol.

Finally, it is unfortunate that the reviewer supports the rationale for their proposed overadjustment by referring to two papers that have shown an apparent heterogeneity of treatment effect by some baseline clinical features, despite the fact that they are methodologically weaker than our study (as partly acknowledged by the reviewer). For instance, we and others have previously commented on some of the limitations of the paper by Brunström (see <https://ebm.bmj.com/content/ebmed/early/2018/05/24/bmjebm-2018-110934.full.pdf> <https://jamanetwork.com/journals/jamainternalmedicine/article-abstract/2677027>). More recently, the central findings of that study were refuted by us using the largest IPD meta-analysis ever conducted to date <https://www.escardio.org/The-ESC/Press-Office/Press-releases/Blood-pressure-lowering-is-even-more-beneficial-than-previously-thought>). In brief, this study with its more rigorous study design did not suggest that CVD status or baseline BP modify the effect of antihypertensive treatment on CVD outcomes. This might also explain why the adjustments proposed by the reviewer had little effect on the findings.

We have checked our manuscript to ensure that the wording is consistent with our assumptions. More specifically, we avoid referring to AF as an independent condition but to patients with AF status at baseline who are expected to have differing comorbidities and characteristics from those without AF. The additional analyses have been reported in the methods section, results and the table added to the supplement.

**Table S11**. Sensitivity analysis for the effect of blood pressure-lowering treatment on primary and secondary outcomes, stratified by the presence of atrial fibrillation at baseline after adjustment for baseline systolic BP, CVD status and diabetes status at baseline:

|  | Intervention | | Comparator | | HR* 95%CI |
| --- | --- | --- | --- | --- | --- |
|  | **Event** | **Total** | **Event** | **Total** |  |
| Major cardiovascular events |  |  |  |  |  |
| AF | 1837 | 6837 | 1837 | 6429 | 0.91 (0.83 to 1.00) |
| No AF | 10752 | 90869 | 10628 | 84435 | 0.92 (0.89 to 0.95) |
| Overall | 12589 | 97706 | 12465 | 90864 | 0.92 (0.89 to 0.95) |
| Stroke |  |  |  |  |  |
| AF | 598 | 6837 | 622 | 6429 | 0.83 (0.72 to 0.97) |
| No AF | 3667 | 90869 | 3759 | 84435 | 0.85 (0.81 to 0.90) |
| Overall | 4265 | 97706 | 4381 | 90864 | 0.85 (0.81 to 0.90) |
| Ischaemic heart disease |  |  |  |  |  |
| AF | 314 | 6837 | 296 | 6429 | 0.91 (0.76 to 1.09) |
| No AF | 5017 | 90869 | 4898 | 84435 | 0.93 (0.89 to 0.98) |
| Overall | 5331 | 97706 | 5194 | 90864 | 0.93 (0.89 to 0.97) |
| Heart failure |  |  |  |  |  |
| AF | 698 | 6748 | 730 | 6346 | 0.89 (0.76 to 1.05) |
| No AF | 2397 | 76897 | 2389 | 70504 | 0.99 (0.92 to 1.07) |
| Overall | 3095 | 83645 | 3119 | 76850 | 0.96 (0.90 to 1.02) |
| Cardiovascular death |  |  |  |  |  |
| AF | 865 | 6837 | 834 | 6429 | 0.90 (0.79 to 1.02) |
| No AF | 2134 | 90311 | 1896 | 83882 | 0.95 (0.89 to 1.01) |
| Overall | 2999 | 97148 | 2730 | 90311 | 0.95 (0.90 to 1.00) |
| All-cause death |  |  |  |  |  |
| AF | 1471 | 6837 | 1335 | 6429 | 1.00 (0.91 to 1.11) |
| No AF | 8641 | 90869 | 7904 | 84435 | 0.98 (0.95 to 1.02) |
| Overall | 10112 | 97706 | 9239 | 90864 | 0.99 (0.96 to 1.02) |

* Standardised by 5-mmHg reduction in systolic blood pressure

5)            Again, the argument that population/setting is accounted for by different baseline hazard functions depends on the assumption that no trial or population characteristics interact with the treatment effect. This, quite simplistic, view is also reflected in the argument concerning fixed- and random-effects model. However, I do think that the sensitivity analysis using both types of models is reassuring, as expected when statistical heterogeneity is low. Results from the I2-analyses and the sensitivity analysis should be presented to the readers in the appendix and commented in the article.

**Reply**: We have now reported all the related details in supplementary Table S8 (shown below) and added a brief comment about the result in the main paper.

Table S8. Fixed and random effects two-stage meta-analyses

| Two-stage model using fixed effect meta-analysis | HR* | 95% CI |
| --- | --- | --- |
| Atrial fibrillation (I^2^ = 24%, p = 0.20) | 0.94 | [0.84 to 1.05] |
| No atrial fibrillation (I^2^ = 28%, p = 0.15) | 0.92 | [0.89 to 0.94] |
| All (I^2^ = 37%, p = 0.08) | 0.92 | [0.89 to 0.94] |
| Two-stage model using random effects meta-analysis |  |  |
| Atrial fibrillation (I^2^ = 19%, p = 0.20) | 0.91 | [0.79 to 1.06] |
| No atrial fibrillation (I^2^ = 40%, p = 0.15) | 0.91 | [0.87 to 0.95] |
| All (I^2^ = 45%, p = 0.08) | 0.91 | [0.87 to 0.95] |

* Standardised by 5-mmHg reduction in systolic blood pressure

6)            Ok, needs to be included in the appendix.

**Reply**: The results are reported in supplementary Table S9.

7)            Great, but the conclusion in the main article needs to be toned down as well. "…even when baseline BP is below recommended treatment thresholds" should be removed or rephrased. Although the analysis >/< 140 mm Hg did not find evidence for an interaction, the analysis had low power and the results in each group was not significant on its own. Phrasing in abstract "no evidence that treatment effects varied according to baseline systolic BP or drug class" is more correct.

**Reply:** The mentioned sentences have been reworded as follows:

*“In conclusion, this study demonstrated that BP-lowering treatment reduces the risk of major cardiovascular events in patients with AF to a similar extent to that of patients without AF, with*

*no evidence that treatment effects vary according to baseline systolic BP or drug class. Owing to their higher absolute cardiovascular risk, treatment in patients with AF is likely to result in greater absolute risk reduction than in patients without AF. Guidelines should be updated to give clear recommendations for pharmacological BP lowering for the prevention of cardiovascular events in patients with AF.”*

One additional minor note is that the Ettehad reference now appears twice.

**Reply:** Thank you for your point. We have corrected this issue.

Reviewer #3: No additional comments

**Reply:** Thank you
