# Supplementary material for: Blood pressure-lowering treatment for the prevention of cardiovascular events in patients with atrial fibrillation: An individual participant data meta-analysis
Source: PLoS Med. 2021 Jun 1;18(6):e1003599. doi: 10.1371/journal.pmed.1003599 (PMC8168843; doi:10.1371/journal.pmed.1003599)
Supplement: Attachment — Submitted filename: Response.docx [file pmed.1003599.s020.docx]

1. Thank you for providing a Financial Disclosure statement in the submission form. This statement is published with the article, therefore should be completed in full sentences and contain the exact wording you wish to be published.

i) State the initials, alongside each funding source, of each author to receive each grant.

Reply: We have added the below complete statements:

“The following authors are supported by grants from the British Heart Foundation “ACPG & KR (grant number: FS/19/64/34673, KR & DC (grant number: PG/18/65/33872, MN & KR (grant number FS/19/36/34346; KR is also in receipt of funding from the UKRI’s Global Challenges Research Fund, Grant Ref ES/Po11055/1, Oxford NIHR Biomedical Research Centre and the Oxford Martin School, University of Oxford.

The funders had no role in study design, data collection and analysis, decision to publish, or preparation of the manuscript.”

ii) State what role the funders took in the study (in the submission form, not the manuscript). If the funders had no role in your study, please state: “The funders had no role in study design, data collection and analysis, decision to publish, or preparation of the manuscript.”

Reply: We have added the below statement:

“The funders had no role in study design, data collection and analysis, decision to publish, or preparation of the manuscript.”

2.Please ensure that the funders and grant numbers match between the Financial Disclosure field and the Funding Information tab in your submission form. Note that the funders must be provided in the same order in both places as well.

Reply: We have revised the mentioned section in the submission form.

3. Please declare all competing interests beginning with the statement "I have read the journal's policy and the authors of this manuscript have the following competing interests:" on the submission form (not the manuscript).

Reply: We have revised the mentioned section in the submission form.

4. We noticed you have 'Other' files in the File Inventory. This file type can't be seen by reviewers. If you would like the reviewers to see these files please change the file type to ‘Supporting Information’.

Reply: We have re-uploaded the mentioned files in the submission form.

5. Please provide all main figures in .tif or .eps format in the File Inventory. Please remove the embedded figures from your manuscript. For more information about how to convert your figure files please see our guidelines: http://journals.plos.org/plosbiology/s/figures. Once you've converted your files to .tif or .eps, please also make sure that your figures meet our format requirements: http://journals.plos.org/plosmedicine/s/figures#loc-file-requirements

Reply: We have uploaded each figure separately as EPS file according to journal instruction.

6. Please list supporting figures as file type Supporting Information.

Reply: Done

7. We noticed you have some occurrences of overlapping text with the following previous publication, which needs to be addressed: https://doi.org/10.1093/ehjci/ehaa946.0672

We understand that some text overlap with your previous work may be unavoidable. However, to avoid copyright issues with the other publisher, in your revision we would ask that ensure you cite the relevant work clearly, and quote or rephrase any duplicated text used (sections of the Abstract, and the Conclusion at lines 344-350).

Reply: Thank you for your comment. The mentioned document is only published ESC congress abstract related to this paper but not any previously published “original” paper. If you still believe that we should change the overlap between congress abstract and the current full paper, we happy to do so.
