# Supplementary material for: Blood pressure-lowering treatment for the prevention of cardiovascular events in patients with atrial fibrillation: An individual participant data meta-analysis
Source: PLoS Med. 2021 Jun 1;18(6):e1003599. doi: 10.1371/journal.pmed.1003599 (PMC8168843; doi:10.1371/journal.pmed.1003599)
Supplement: Attachment — Submitted filename: Requests from Editors.docx [file pmed.1003599.s021.docx]

Requests from Editors:

1. Similarity with published abstract: It appears that there is some overlap between the content of the manuscript and your published abstract. Regarding the text overlap, please slightly reword any directly replicated text, if possible, to avoid copyright issues.
For example:

In the abstract: "The hazard ratio for major cardiovascular events was 0.91 in patients with AF (95% confidence interval 0.83 to 1.00) and 0.91 without AF (95% confidence interval 0.88 to 0.93) for each 5-mmHg reduction in systolic BP, with no difference between subgroups. In patients with AF, there was no evidence that treatment effects varied according to baseline systolic BP or drug class."
The final sentence of the Discussion/Conclusion: "Guidelines should be updated to give clear recommendations for pharmacological BP lowering for prevention of cardiovascular events in patients with AF."

**Re**: We have revised the mentioned parts of the manuscript.

In addition, Figure 4 is reproduced from the published abstract: (https://doi.org/10.1093/ehjci/ehaa946.0672). If you would like to include the figure in the manuscript, please contact OUP to obtain their agreement to it being published under our license. An alternative would be to remove the figure and present the data in a different format (e.g. as a table).
**Re**: Please find attached the agreement copyright license.

2. Data Availability Statement: Thank you for your response "We have now added the data availability statement as requested and explained how the interested authors could ask for BPLTTC dataset." Please update the data availability statement of the manuscript submission form with the information provided on Page 16 Lines 363-366.

**Re**: Thank you. Done.

3. Please remove the highlighting in the final "clean" version submitted.
**Re**: Done

4. Methods: Page 6: Lines 126-127: Please provide a reference to the supporting information file for the protocol (e.g. S1_Protocol).
**Re**: Done

5. Discussion: Line 302: Please revise this sentence, as there may be a word missing here: "...ideal SBP target for prevention of cardiovascular was found..."
**Re**: We have revised the mentioned parts of the manuscript.

6. Page 16: Please remove the Financial Disclosure and Data Availability Statement sections from the main text, and ensure the information is entered in the appropriate section of the manuscript submission form.
**Re**: Done

7. References: Please double check the formatting of references, using NLM journal title abbreviations where appropriate (e.g. JAMA Intern Med instead of JAMA Internal Medicine). Please use the "Vancouver" style for reference formatting, and see our website for other reference guidelines: https://journals.plos.org/plosmedicine/s/submission-guidelines#loc-references
**Re**: We have now revised the reference style.

Any attachments provided with reviews can be seen via the following link:
